# Corilagin Counteracts IL-13Rα1 Signaling Pathway in Macrophages to Mitigate Schistosome Egg-Induced Hepatic Fibrosis

**DOI:** 10.3389/fcimb.2017.00443

**Published:** 2017-10-18

**Authors:** Yi-Qing Li, Yun-Fei Chen, Yi-Ping Dang, Yao Wang, Zhen-Zhong Shang, Qian Ma, Yu-Jie Wang, Juan Zhang, Lei Luo, Quan-Qiang Li, Lei Zhao

**Affiliations:** ^1^Department of Vascular Surgery, Union Hospital, Tongji Medical College, Huazhong University of Science and Technology, Wuhan, China; ^2^School of Clinical Medicine, Hubei University of Chinese Medicine, Wuhan, China; ^3^School of Basic Medical Sciences, Guangxi University of Chinese Medicine, Nanning, China; ^4^School of Life Science, Hubei University, Wuhan, China; ^5^School of Clinical Medicine, Guangxi University of Chinese Medicine, Nanning, China; ^6^Department of Infectious Diseases, Union Hospital, Tongji Medical College, Huazhong University of Science and Technology, Wuhan, China

**Keywords:** corilagin, liver fibrosis, schistosome egg, IL-13Rα1, M2 macrophage

## Abstract

The IL-13Rα1 signaling pathway and M2 macrophages play crucial roles in schistosome egg-induced hepatic fibrosis via the expression of pro-fibrotic molecules. This study aims to investigate the inhibitory effect and mechanism of action of corilagin on schistosome egg-induced hepatic fibrosis via the IL-13Rα1 signaling pathway in M2 macrophages *in vitro* and *in vivo*. The mRNA and protein expression of IL-13Rα1, PPARγ, KLF4, SOCS1, STAT6, p-STAT6, and TGF-β was measured *in vitro* with corilagin treatment after IL-13 stimulation and *in vivo* corilagin treatment after effectively killing the adult schistosomes in schistosome-infected mice. Histological analysis of liver tissue was assessed for the degree of hepatic fibrosis. The results revealed that corilagin significantly reduced the expression of PPARγ, KLF4, SOCS1, p-STAT6, and TGF-β compared with model group and praziquantel administration (*p* < 0.01 or *p* < 0.05) *in vivo* and *in vitro*, which indicated a strong inhibitory effect of corilagin on IL-13Rα1 signaling pathway. As well, the inhibitory effect of corilagin showed a significant dose-dependence (*p* < 0.05). The area of fibrosis and distribution of M2 macrophages in mouse liver tissue were reduced significantly and dose-dependently with corilagin treatment compared to model group or praziquantel administration (*p* < 0.01 or *p* < 0.05), indicating that corilagin suppressed IL-13Rα1 signaling pathway and M2 macrophage polarization effectively *in vivo*. Furthermore, the anti-fibrogenic effect persisted even when IL-13Rα1 was up- or down-regulated *in vitro*. In conclusion, corilagin can suppress schistosome egg-induced hepatic fibrosis via inhibition of M2 macrophage polarization in the IL-13Rα1 signaling pathway.

## Introduction

Schistosomiasis is an ancient parasitic disease that imposes a considerable economic and health burden, disabling more than it kills. As of 2014, over 207 million people were infected with schistosoma, and approximately 700 million people were at risk of infection (Shaker et al., 2014). Schistosomiasis is endemic in China, and China is one of the primary epidemic areas of *Schistosoma japonicum* (Colley et al., 2014; Song and Wu, 2015). Schistosomiasis hepatic fibrosis is a chronic immune disease caused by schistosome eggs that can lead to hepatosplenomegaly, ascites, portal hypertension and sometimes death as a result of upper gastrointestinal hemorrhage or hepatic encephalopathy.

Fibrosis is a component of normal tissue repair after acute inflammatory injury. However, it causes irreversible tissue damage in the case of chronic inflammation. Schistosomiasis hepatic fibrosis results from the soluble egg antigen (SEA) secreted by mature schistosome eggs, which induces the alternative activation of macrophages (M2 macrophages) that play a vital role in fibrogenesis (Martinez et al., 2009). In the acute phase, M2 macrophages can reduce the production of proinflammatory cytokines and prevent the induction of acute inflammatory lesions by eggs (Herbert et al., 2004). However, in the chronic phase, M2 macrophages secrete pro-fibrotic cytokines, leading to hepatic fibrosis (Burke et al., 2009). Liver macrophages (Kupffer cells) are the major contributors to schistosome granuloma (Xie et al., 2015). After the SEA is released, the host immune response is polarized into a Th2-biased response, with increased expression of IL-13 (Pearce and MacDonald, 2002). IL-13 was demonstrated to be detrimental in schistosomiasis (Fallon et al., 2000). And it is a key cytokine which strongly induced the expression of pro-fibrotic cytokines and M2 macrophage polarization via the IL-13α1 signaling pathway (Liu et al., 2012; Sica and Mantovani, 2012; Song and Wu, 2015). IL-13 binding the IL-4Rα/IL-13Rα1 receptor complex triggers signal transduction and increases the expression of PPARγ, KLF4 and SOCS1 (Biswas et al., 2012; Sica and Mantovani, 2012). These M2 cytokines stimulate the Kupffer cells and hepatic stellar cells to produce TGF-β, a crucial factor in fibrosis, and accelerate fibrosis progression (Fabregat et al., 2016). Thus, inhibition of M2 polarization of macrophages and IL-13Rα1 signaling pathway may be the key to suppressing schistosomiasis hepatic fibrosis.

Currently, the most widely used drug for the treatment of schistosomiasis is praziquantel, which can kill the mature parasite but has little effect on schistosome eggs (Gryseels et al., 2006). Studies have shown that schistosomiasis hepatic fibrosis and granulomatous inflammatory disease continue even after effective anti-*Schistosoma* treatment (Cioli and Pica-Mattoccia, 2003; Southgate et al., 2005; Gryseels et al., 2006). Resistance to praziquantel has also been reported (Ross et al., 2002; Burke et al., 2009). Therefore, suppressing fibrosis and granuloma formation after effective killing of mature *Schistosoma* has become a key issue in the treatment of chronic and advanced schistosomiasis (Chu et al., 2011). Many researchers have tried to find new therapeutics to inhibit schistosomiasis hepatic fibrosis.

Corilagin (C_27_H_22_O_18_) is an active component of many medicinal plants, with a variety of pharmacological activities including anti-tumor, anti-oxidative, anti-atherogenic, thrombolytic, anti-hypertensive, hepatoprotective, antiviral, antibacterial and anti-inflammatory effects (Zhao et al., 2008; Guo et al., 2010, 2015a; Jin et al., 2013; Li et al., 2017). Its known chemical structure was presented in our previously published paper (Zhao et al., 2008).

We previously confirmed the anti-fibrogenic effect of corilagin (Huang et al., 2013; Yang et al., 2016) and investigated intervention with the IL-13R signaling pathway in HSC (Li et al., 2016) and a mouse model without praziquantel treatment (Du et al., 2016; Yang et al., 2016). In this study, IL-13 was used *in vitro* to stimulate Ana-1 cells to simulate egg-induced fibrosis. For the *in vivo* study, praziquantel was administered after the mice were infected by the cercaria to kill the adult schistosomes. We investigated the inhibitory effect of corilagin on the molecules involved in IL-13Rα1 signaling pathway (PPARγ, KLF4, SOCS1 and p-STAT6) and egg-induced fibrogenesis (TGF-β) *in vitro* and *in vivo*. Furthermore, we observed the fibrosis degree and distribution of M2 macrophages in schistosomiasis mouse liver by the method of histology and immunohistochemistry in animal model. Through this study, we aimed to develop a new strategy for the treatment of schistosomiasis hepatic fibrosis which praziquantel had little effect on.

## Materials and methods

Chemicals and reagents could be seen in the Supplementary Data Sheet 2.

### Cell culture

Ana-1 cell line is a well-established murine macrophage cell line, and it can be transformed into either M1 or M2 macrophage with some administration (Chen et al., 2012). It was purchased from the Type Culture Collection of the Chinese Academy of Sciences, Shanghai, China. The cells were cultured in RPMI-1640 medium containing 10% fetal bovine serum in an incubator at 37°C, 5% CO_2_ and saturated humidity.

### Corilagin cytotoxicity assays

According to the previously published paper (Li et al., 2016), we evaluated cell viability using the CCK8 assay. The Ana-1 cells were plated at a density of 1 × 10^4^/mL cells in 96-well culture plates with different concentrations of corilagin (400, 200, 100, 50, and 25 mg/mL). The Ana-1 cells without corilagin administration was set as a positive control, and a blank well-containing the medium culture was set as a negative control. After 24 h, the cell morphology was observed, and 10 μl of CCK-8 solution was added to each well, which was then incubated for 2 h before the absorbance was measured at 450 nm using a microplate reader. Three wells in each group were measured.

### *In vitro* corilagin treatment after Il-13 stimulation

Recombinant IL-13 was diluted to 5 μg/ml with RPMI-1640 medium containing 5% fetal bovine serum and stored at −20°C. Praziquantel was diluted to 5 pg/ml with PBS and stored at 4°C. Corilagin was diluted to 2 mg/ml with PBS and stored at 4°C. To explore the anti-fibrogenic effect of corilagin with the praziquantel intervention via the IL-13α1 signaling pathway, Ana-1 cells were divided into six groups. The high- (100 μg/ml), medium- (50 μg/ml), and low-concentration (25 μg/ml) corilagin groups were stimulated with rIL-13 (50 ng/ml) for 24 h, followed by treatment of corilagin for 24 h, and no praziquantel was administered. The praziquantel group was stimulated with rIL-13 (50 ng/ml) for 24 h, followed by treatment of praziquantel (5 pg/ml) for 24 h. The model group was incubated with rIL-13 (50 ng/ml) only. The untreated group was the control group.

### IL-13Rα1 up-regulation by lentivirus transfection

IL-13Rα1 was up-regulated by means of lentivirus transfection containing pre-IL-13Rα1 and green fluorescent protein. Ana-1 cells were cultured in enhanced infection solution with the vehicle for 96 h at a MOI of 40. The transfection efficiency was observed under a fluorescence microscope (Olymbus IX2 series microscope). To further confirm the effect of corilagin on the IL-13α1 pathway, Ana-1 cells were divided into seven groups for an up-regulation experiment. The group stimulated with rIL-13 alone without IL-13Rα1 up-regulation was included in the previous experiments. Therefore, we set up this part of the experiment without the group of rIL-13 stimulation alone. In the high- (100 μg/ml), medium- (50 μg/ml), low-concentration (25 μg/ml) corilagin up-regulation groups, the IL-13Rα1 up-regulated Ana-1 cells were stimulated with rIL-13 (50 ng/ml) for 24 h before corilagin was added. In the model up-regulation group, the IL-13Rα1 up-regulated Ana-1 cells were incubated with rIL-13 (50 ng/ml) only. In the up-regulation group, the IL-13Rα1 up-regulated Ana-1 cells were cultured without any treatment. In the empty-vector group, the Ana-1 cells were cultured in enhanced infection solution with the empty vector for 96 h only. The untreated group was the control group.

### IL-13Rα1 down-regulation by siRNA transfection

IL-13Rα1 was down-regulated by means of siRNA transfection. The siRNA was purchased from RiboBio company (Guangzhou, China), and the specificity of siRNA was checked by Primer BLAST. The target sequence of the siRNA was CCTGCACTGGAAGAAGTAT, and the concentration of the siRNA was 0.02 nmol/μl. The siRNA contained cy3 dye. Ana-1 cells were incubated in RPMI-1640 medium containing 5% fetal bovine serum with 0.25% (5 μl Lipofectamine™ 2000 in 2 ml cell culture medium) Lipofectamine™ 2000 (purchased from Invitrogen) and 100 pmol of siRNA for 6 h, then cultured with RPMI-1640 medium containing 5% fetal bovine serum that did not contain siRNA or Lipofectamine™ 2000 for 48 h. Subsequently, the transfection efficiency was observed under a fluorescence microscope. To further confirm the effect of corilagin on the IL-13α1 pathway, Ana-1 cells were divided into seven groups in the down-regulation experiment. In the high- (100 μg/ml), medium- (50 μg/ml), low-concentration (25 μg/ml) corilagin down-regulation groups, the IL-13Rα1 down-regulated Ana-1 cells were stimulated with rIL-13 (50 ng/ml) for 24 h before corilagin was added. In the model down-regulation group, the IL-13Rα1 down-regulated Ana-1 cells were incubated with rIL-13 (50 ng/ml) only and cultured without any treatment. In the empty-vector group, the Ana-1 cells were transfected by the empty vector. The untreated group was the control group.

### Corilagin treatment on mouse schistosomiasis model after praziquantel administration

The animal experiments were approved by the Tongji Medical College, HUST Institutional Animal Care and Use Committee ([2016] IACUC Number: 598). A total of 42 male C57BL/6 mice, 6 to 8 weeks old and weighing 18–22 g, were purchased from the Hubei Provincial Center for Disease Control and Prevention (Wuhan, China). The mice were randomized into seven groups, with six mice per group. The mice were fed an untreated diet and given access to water under standard laboratory conditions at a temperature of 25 ± 2°C, a relative humidity of 50 ± 15% and a normal circadian rhythm (12-h dark/12-h light). All study protocols were conducted in accordance with the Guidelines for the Care and Use of Laboratory Animals of Huazhong University of Science and Technology and approved by the Ethics Committee of Union Hospital, Tongji Medical College, Huazhong University of Science and Technology. The control group served as a blank control without any administration or infection. The animals in all groups except the control group were infected with 25 ± 5 *Schistosoma* japonicum cercaria via the abdominal-patch method (Huang et al., 2013). After anaesthesia with 2% pentobarbital sodium (intraperitoneal injection, 45 mg/kg), the abdominal skin without hair was exposed to the cercaria carried by *Oncomelania hupensis* for 30 min to infect the mice. Four weeks after infection, the animals in all groups except the control group were administered praziquantel orally at 500 mg/kg·d for 5 days to kill the adult schistosomes. Subsequently, the high- (80 mg/kg·d), medium- (40 mg/kg·d), and low-concentration (20 mg/kg·d) corilagin groups were administered corilagin orally for 21 days. The praziquantel group as a positive control continued to be administered praziquantel orally at 500 mg/kg·d for 21 days after the initial 5 days of praziquantel treatment. The levofloxacin group as a negative control was administered levofloxacin orally at 100 mg/kg·d for 21 days. The model infection group was administered with no more drugs after 5-day praziquantel treatment. Finally, the mice were euthanized after anesthetization of 4% chloral hydrate by intraperitoneal injection and the specimens were collected. Blood was collected via retro-orbital route, centrifuged at 3,500 rpm for 10 min and sera were stored at −20°C for ELISA. Part of the liver tissue was fixed in 10% formalin for tissue examination. The rest was stored in liquid nitrogen for PCR and Western-blot assays. During the dissection of mice, the adult schistosomes were not observed in the liver of mice or in the portal system, which indicated the 5-day praziquantel treatment was effective on killing the adult schistosomes.

### Real-time quantitative PCR

The expression of PPARγ, KLF4, SOCS1 and STAT6 was quantified by real-time qPCR. The operation and analysis was performed according to our previously published protocol (Guo et al., 2015b). Total RNA in Ana-1 cells was isolated using RNAiso Plus (TaKaRa Company). The A260/280 ratio of the mRNA was detected by spectrophotometer (UV751GD UV/VIS spectrophotometer, Implen company, German), and only the samples whose A260/280 ratios were between 1.8 and 2.2 were used. The cDNAs were produced with the PrimeScript™ RT reagent kit (TaKaRa company) and incubated at 37°C for 15 min and 85°C for 5 s. Real-time PCR reactions were performed using a StepOne Plus device (Applied Biosystems) at 95°C for 10 s, followed by 40 cycles of 95°C for 5 s and 60°C for 20 s according to the instructions for the SYBR Premix Ex Taq kit (TaKaRa company). The standard curves indicated that all the slopes ranged from −3.3515 to −3.222 and the *R*^2^ values were greater than 0.99. Therefore, the data were analyzed by the 2^−ΔΔCT^ method. The number of PCR cycles varied according to the expression level of the target gene. An appropriate primer concentration and number of cycles was determined to ensure that the PCR was taking place in the linear range and thereby guaranteed a proportional relationship between input RNA and the cycles readout. All primers were synthesized by TSINGKE (Wuhan, China). GAPDH and β-actin were used as reference genes. In our study, the trends of KLF4 expression were consistent in the case of two reference genes (GAPDH and β-actin). Therefore, we chose GAPDH as the reference gene for the following qPCR assay. Three sets of duplicate data were included for statistical analysis. The primers were constructed and purchased from Tsingke Biological Technology Company (Beijing, China). The specificity of primer was checked by Primer Blast. The standard curves and amplification curves could be seen in Supplementary Data Sheet 1.

### Western-blot analysis

The abundance of PPARγ, KLF4, SOCS1, STAT6 and p-STAT6 was detected by Western blot. The operation and analysis were performed as described in a previously published paper (Wang et al., 2017). The protein was separated on SDS-PAGE gels and then transferred to nitrocellulose filter membrane, which was blocked overnight with 5% non-fat milk in TBST. Subsequently, the membrane was probed with the indicated antibody at 4°C before being washed three times in TBST and then incubated with an HRP-labeled secondary antibody. The dilutions of the primary antibodies were as follows: PPARγ (Proteintech Company, Wuhan, China), 1:1,000; KLF4 (Santa Cruz Biotechnology Company, Dallas, TX, US), 1:300; SOCS1 (Abclonal Technology, Woburn, MA, US), 1:500; p-STAT6 (CST, Boston, MA, US), 1:1,000.

### Elisa assay

The TGF-β levels in the cell supernatant or in the sera of the mice were determined by ELISA. The sera were assayed for TGF-β with Mouse TGF-β ELISA kits (Elabscience, Wuhan, China). The procedures were conducted according to the instruction manuals for the kits. Sterile PBS was used as control.

### Histological analysis

Hepatic tissue samples were obtained, formalin-fixed and paraffin-embedded. As in our previously published paper, haematoxylin and eosin (HE) staining (Jin et al., 2015) and Masson's trichrome staining (Li et al., 2016) were performed. Masson's trichrome stains hepatic cells in red and collagen in blue, which could help to clarify the degree of fibrosis. CD206 was a cell surface marker which could distinguish M2 macrophages from M1 macrophages. Immunohistochemistry for CD206 was performed to observe the M2 macrophages distribution in the liver tissue sections. The operation and analysis was performed according to our previously published protocol (Du et al., 2016). CD206 antibody was purchased from Proteintech Group, INC (Chicago, USA), catalog number 18704-1-AP, and its dilution was 1:200. The dilution of CD206 antibody was 1:200. The granuloma area and IOD of CD206 was measured by Imag-Pro Plus 6.0.

### Statistical analysis

The data are presented as the means ± SD. Referring to a previously published statistical approach (Ding et al., 2015), Kolmogorov-Smirnov (K-S) test and Shapiro-Wilk (S-W) test were performed to assess the normalcy. The significance of K-S and S-W test indicated that the data were normally distributed (*p* > 0.05). Levene's test was performed to assess the equality of variances. The significance of Levene's test indicated the variances of the data from different groups were equal (*p* > 0.05). The measurement data were compared between the two groups with Student's *t*-test. Multiple comparisons between multiple groups were performed with one-way ANOVA tests, and a *post-hoc* test was performed by the Student-Newman-Keuls (S-N-K) method. The statistical analyses were conducted with SPSS 12.0 software. Statistical significance was defined at *p* < 0.05.

## Results

### Cytotoxicity of corilagin in ana-1 cells

**Figure 1:**
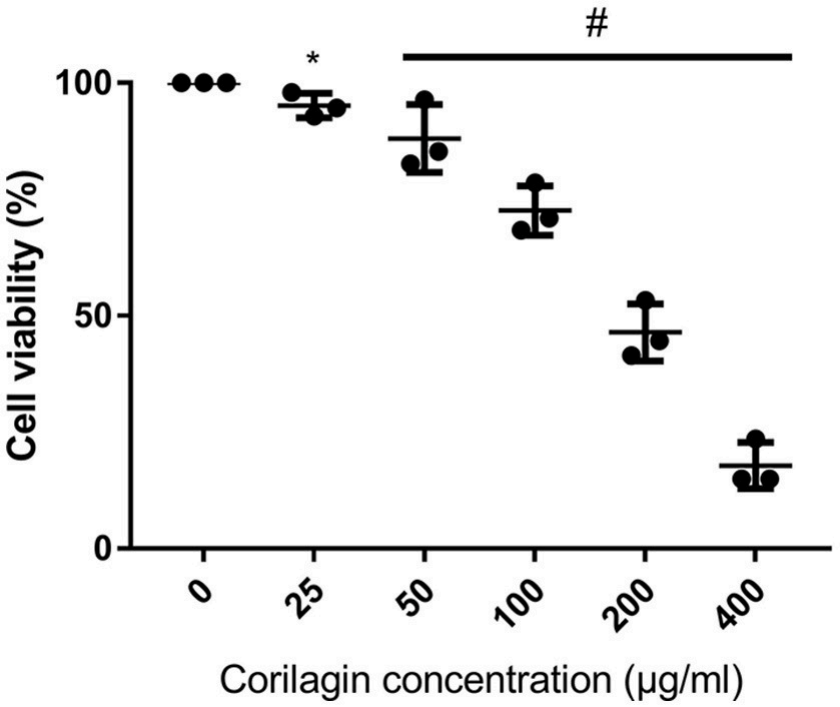
CCK8 assay of Ana-1 cell viability after Corilagin treatment for 24 h. ^#^*p* < 0.05 determined by one-way ANOVA and significant differences from the respective values determined by S-N-K method (*n* = 3). ^*^p < 0.05 compared with the 0 μg/ml group (*n* = 3, student's *t*-test).

The viability of Ana-1 cells with corilagin administration was illustrated in Figure 1. As illustrated, the viabilities of all corilagin groups decreased compared with the 0 μg/ml corilagin group (*p* < 0.05, Student's *t*-test, *n* = 3). In our study, we chose the concentrations which could result in a cell viability more than 70% (100, 50, 25, μg/ml). With the corilagin concentration ranging from 50 to 400 μg/ml, the viability decreased with increasing concentrations of corilagin (*p* < 0.05, one-way ANOVA, *post-hoc*: S-N-K method, *n* = 3), suggesting a dose-dependent effect.

### Effect of corilagin on IL-13Rα1 signaling pathway after IL-13 stimulation in ana-1 cells

**Figure 2:**
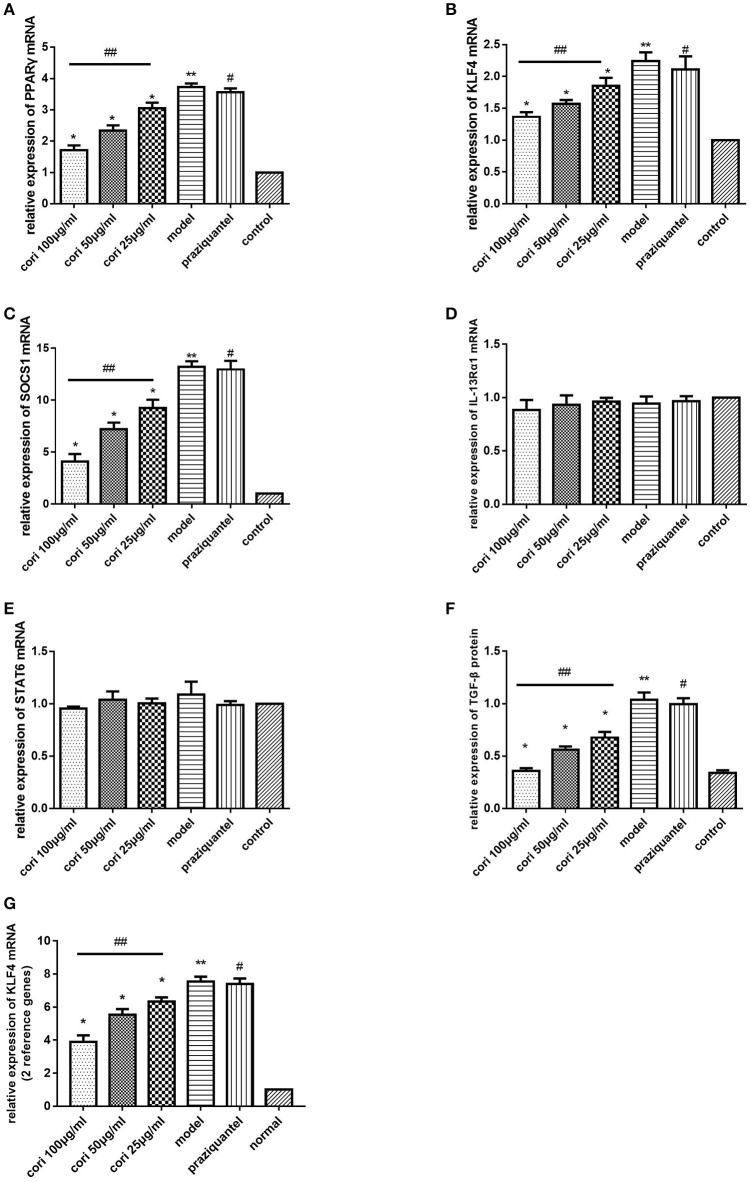
Effect of Corilagin on mRNA expression of PPARγ, KLF4, SOCS1, IL-13Rα1, STAT6, and protein expression of TGF-β after IL-13 stimulation in Ana-1 cells. Ana-1 cells were stimulated with rIL-13 for 24 h except control group. Subsequently, the cells were administrated with corilagin or praziquantel for 24 h respectively. Corilagin showed a dose-dependent inhibitory effect on IL-13Rα1 signaling pathway after IL-13 stimulation. **(A)** The PPARγ mRNA expression levels detected by real-time qPCR. ^*^p < 0.01 compared with the model group (*n* = 3, student's *t*-test); ^**^p < 0.01 compared with the control group (*n* = 3, student's *t*-test); #*p* < 0.01 compared with the control group (*n* = 3, student's *t*-test); ##*p* < 0.05 determined by one-way ANOVA and significant differences from the respective values determined by S-N-K method (*n* = 3). **(B)** The KLF4 mRNA expression levels detected by real-time qPCR. ^*^p < 0.05 compared with the model group (*n* = 3, student's *t*-test); ^**^p < 0.01 compared with the control group (*n* = 3, student's *t*-test); #*p* < 0.01 compared with the control group (*n* = 3, student's *t*-test); ##*p* < 0.05 determined by one-way ANOVA and significant differences from the respective values determined by S-N-K method (*n* = 3). **(C)** The SOCS1 mRNA expression levels detected by real-time qPCR. ^*^p < 0.01 compared with the model group (*n* = 3, student's *t*-test); ^**^p < 0.01 compared with the control group (*n* = 3, student's *t*-test); #*p* < 0.01 compared with the control group (*n* = 3, student's *t*-test); ##*p* < 0.05 determined by one-way ANOVA and significant differences from the respective values determined by S-N-K method (*n* = 3). **(D)** The IL-13Rα1 mRNA expression levels detected by real-time qPCR. It was shown that no difference among all groups. **(E)** The STAT6 mRNA expression levels detected by real-time qPCR. It was shown that no difference among all groups. **(F)** Effect of Corilagin on TGF-β protein expression detected by ELISA. ^*^p < 0.01 compared with the model group (*n* = 3, student's *t*-test); ^**^p < 0.01 compared with the control group (*n* = 3, student's *t*-test); #*p* < 0.01 compared with the control group (*n* = 3, student's *t*-test); ##*p* < 0.05 determined by one-way ANOVA and significant differences from the respective values determined by S-N-K method (*n* = 3). **(G)** The KLF4 mRNA expression levels with two reference genes detected by real-time qPCR. ^*^p < 0.05 compared with the model group (*n* = 3, student's *t*-test); ^**^p < 0.01 compared with the control group (*n* = 3, student's *t*-test); #*p* < 0.01 compared with the control group (*n* = 3, student's *t*-test); ##*p* < 0.05 determined by one-way ANOVA and significant differences from the respective values determined by S-N-K method (*n* = 3). Data are shown with mean with SD.

**Figure 3:**
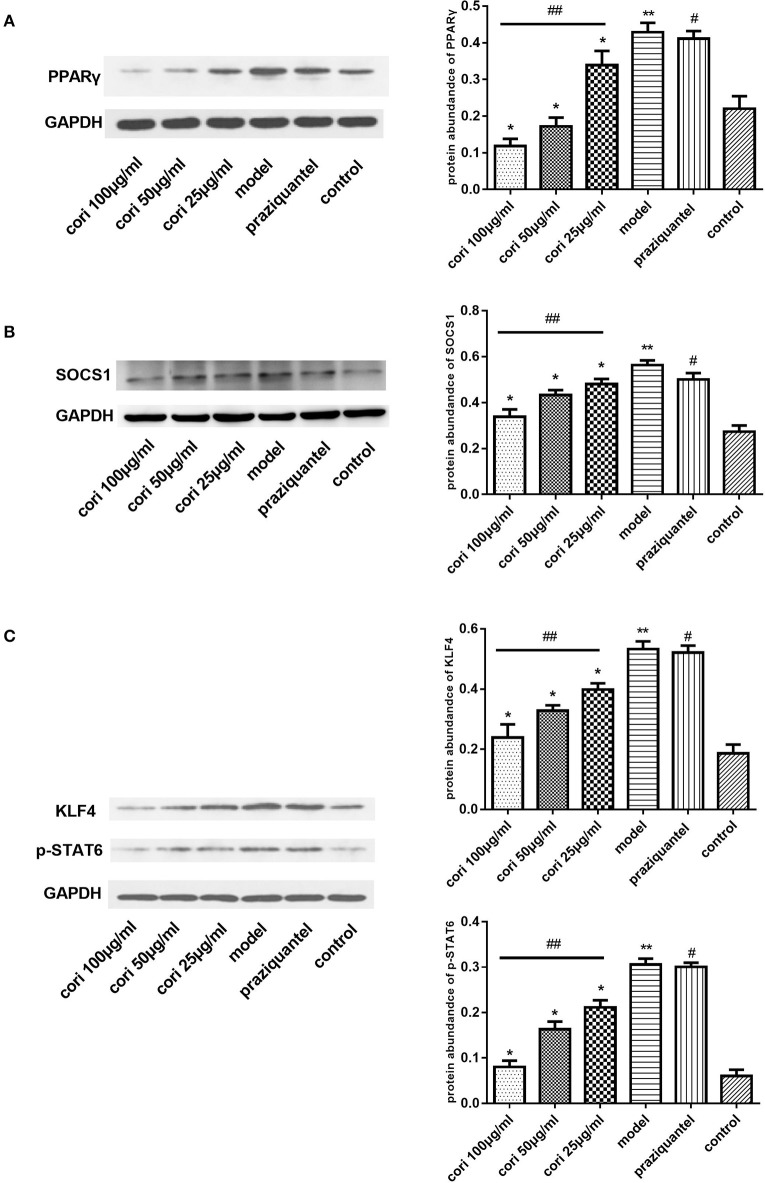
Effect of Corilagin on protein abundance of PPARγ, KLF4, SOCS1, and p-STAT6 after IL-13 stimulation in Ana-1 cells. Ana-1 cells were stimulated with rIL-13 for 24 h except control group. Subsequently, the cells were administrated with corilagin or praziquantel for 24 h respectively. Corilagin showed a dose-dependent inhibitory effect on IL-13Rα1 signaling pathway after IL-13 stimulation. **(A)** The PPARγ protein abundance detected by western blot assay. ^*^p < 0.01 compared with the model group (*n* = 3, student's *t*-test); ^**^p < 0.01 compared with the control group (*n* = 3, student's *t*-test); #*p* < 0.01 compared with the control group (*n* = 3, student's *t*-test); ##*p* < 0.05 determined by one-way ANOVA and significant differences from the respective values determined by S-N-K method (*n* = 3). **(B)** The SOCS1 protein abundance detected by western blot assay. ^*^p < 0.01 compared with the model group (*n* = 3, student's *t*-test); ^**^p < 0.01 compared with the control group (*n* = 3, student's *t*-test); #*p* < 0.01 compared with the control group (*n* = 3, student's *t*-test); ##*p* < 0.05 determined by one-way ANOVA and significant differences from the respective values determined by S-N-K method (*n* = 3). **(C)** The KLF4 and p-STAT6 protein abundance detected by western blot assay. ^*^p < 0.01 compared with the model group (*n* = 3, student's *t*-test); ^**^p < 0.01 compared with the control group (*n* = 3, student's *t*-test); #*p* < 0.01 compared with the control group (*n* = 3, student's *t*-test); ## *p* < 0.05 determined by one-way ANOVA and significant differences from the respective values determined by S-N-K method (*n* = 3). Data are shown with mean with SD.

PPARγ, KLF4, SOCS1, and p-STAT6 are important regulators of M2 macrophage polarization in the IL-13α1 signaling pathway (Sica and Mantovani, 2012). Therefore, we investigated the expression of genes besides IL-13Rα1 to explore the anti-fibrogenic effect of corilagin. As illustrated in Figure 2, the mRNA levels of PPARγ, KLF4 and SOCS1 were detected by real-time qPCR (Figures 2A–C,G), and the TGF-β levels in the cell supernatant were detected by ELISA (Figure 2F). Meanwhile, we detected mRNA levels of IL-13Rα1 and STAT6 by real-time qPCR (Figures 2D,E). The abundance of PPARγ, KLF4, SOCS1, and p-STAT6 proteins was detected by Western blot (Figure 3). The levels of PPARγ, KLF4, SOCS1, and p-STAT6 in the model experimental groups were increased compared with levels in the control group (*p* < 0.01, Student's *t*-test, *n* = 3), which revealed the model is successful and the expression of these molecules increased with IL-13 stimulation. Meanwhile, with corilagin treatment after IL-13 stimulation, the levels of these molecules decreased significantly compared with the levels in the model group (*p* < 0.01 or 0.05, Student's *t*-test, *n* = 3). And the levels decreased with increasing concentrations of corilagin (*p* < 0.05, one-way ANOVA, *post-hoc*: S-N-K method, *n* = 3), which confirmed a dose-dependent inhibitory effect of corilagin on the cytokines in IL-13Rα1 signaling pathway *in vitro*. With praziquantel treatment after IL-13 stimulation, the levels of these molecules showed no significant difference compared with the model group (*p* > 0.05, Student's *t*-test, *n* = 3), which indicated praziquantel had little effect on the cytokines in IL-13Rα1 signaling pathway. There were no significant differences in the levels of IL-13Rα1 and STAT6 among groups (*p* > 0.05). As illustrated in Figures 2B,G, the trends of KLF4 mRNA expression were showed to be consistent when real-time qPCR was performed with two reference genes in our study.

### Effect of up-regulation of IL-13Rα1 via lentivirus transfection

**Figure 4:**
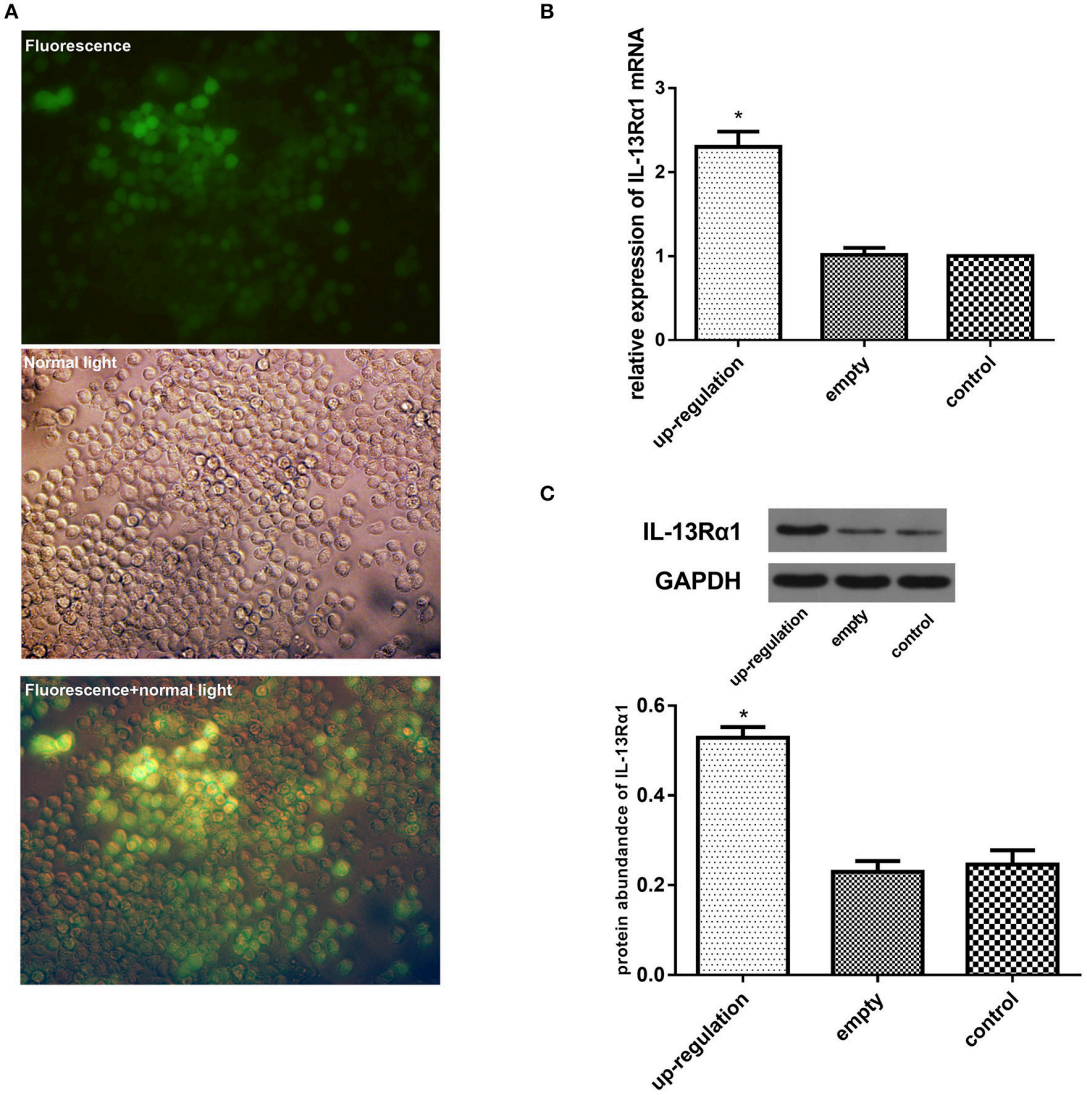
Effect of up-regulation of IL-13Rα1 via lentivirus transfection. Ana-1 cells were infected with lentivirus or empty vector for 96 h except control group. The expression of IL-13Rα1 was increased significantly with lentivirus transfection. **(A)** Effect of lentivirus transfection: 96 h after the ana-1 cells were infected by lentivirus, the cells were observed in the fluorescence, in control light and in both the fluorescence and control light respectively. **(B)** Effect of lentivirus transfection on IL-13Rα1 mRNA levels detected by real-time qPCR. ^*^p < 0.01 compared with the empty and control group (*n* = 3, student's *t*-test). **(C)** Effect of lentivirus transfection on IL-13Rα1 protein abundance detected by western blot assay. ^*^p < 0.01 compared with the empty and control group (*n* = 3, student's *t*-test). Data are shown with mean with SD.

To further explore the anti-fibrotic effect of corilagin in hepatic schistosomiasis, we up-regulated the expression of IL-13Rα1 by lentivirus transfection. The transfection effect was observed under a fluorescence microscope and examined by real-time qPCR, ELISA and Western blot. The Ana-1 cells were observed under a fluorescence microscope 96 h after lentivirus transfection (Figure 4A). The mRNA levels of IL-13α1 were determined by real-time qPCR, and the expression of IL-13Rα1 in up-regulation group increased by 130% compared to the control group (Figure 4B). The abundance of IL-13α1 protein was determined by Western blot, and the abundance of IL-13Rα1 protein in up-regulation group increased by 115% compared to the control group (Figure 4C). As illustrated, the fluorescence was strong and most cells were infected successfully. The levels of IL-13Rα1 mRNA and protein in the up-regulation group were increased significantly compared with the control groups (*p* < 0.01, Student's *t*-test, *n* = 3). The levels in the empty-vector group showed no significant difference compared to the control group (*p* > 0.05, Student's *t*-test, *n* = 3).

### Effect of down-regulation of IL-13Rα1 via siRNA transfection

**Figure 5:**
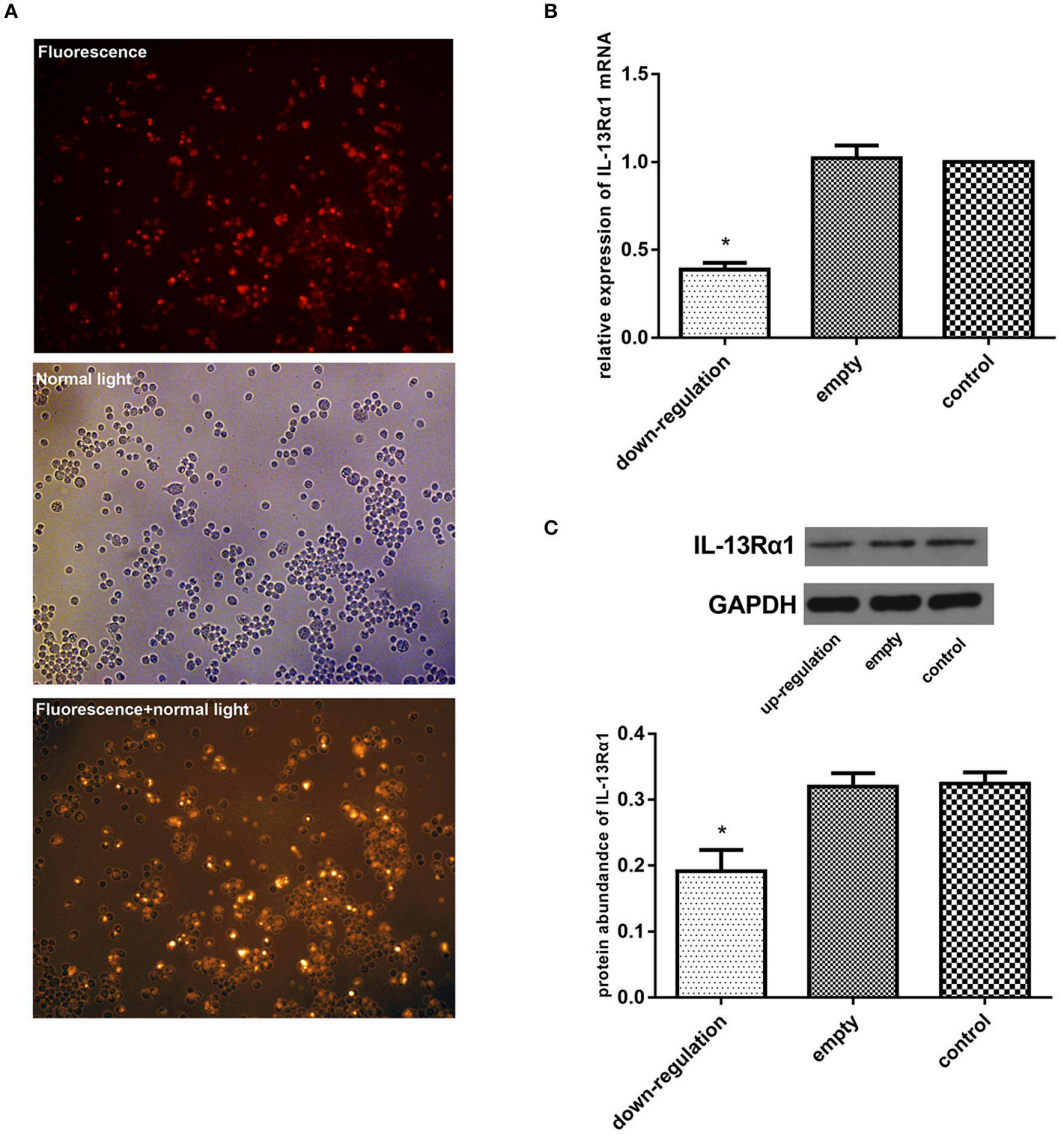
Effect of down-regulation of IL-13Rα1 via siRNA transfection. The expression of IL-13Rα1 was decreased significantly with siRNA transfection. Ana-1 cells were infected with IL-13Rα1 siRNA or empty vector for 48 h except control group. **(A)** Effect of siRNA transfection: 48 h after the ana-1 cells were infected, the cells were observed in the fluorescence, in control light and in both the fluorescence and control light respectively. **(B)** Effect of siRNA transfection on IL-13Rα1 mRNA levels detected by real-time qPCR. ^*^p < 0.01 compared with the empty and control group (*n* = 3, student's *t*-test). **(C)** Effect of siRNA transfection on IL-13Rα1 protein abundance detected by western blot assay. ^*^p < 0.01 compared with the empty and control group (*n* = 3, student's *t*-test). Data are shown with mean with SD.

We down-regulated the expression of IL-13Rα1 by siRNA transfection. The transfection effect was observed under a fluorescence microscope and examined by real-time qPCR, ELISA and Western blot. The Ana-1 cells were observed under a fluorescence microscope 48 h after the siRNA transfection (Figure 5A). As illustrated, the fluorescence was strong and most cells were infected successfully. The mRNA levels of IL-13α1 were determined by real-time qPCR, and the expression of IL-13Rα1 in down-regulation group dropped 61% compared to the control group (Figure 5B). The abundance of IL-13α1 was determined by Western blot, and the abundance of IL-13Rα1 in down-regulation group dropped 41% (Figure 5C). As shown in Figure 5, the level of IL-13Rα1 in the down-regulation group was decreased significantly compared with the empty-vector and control groups (*p* < 0.01, Student's *t*-test, *n* = 3). The level in the empty-vector group showed no significant differences compared with that in the control group (*p* > 0.05, Student's *t*-test, *n* = 3).

### Effect of corilagin on the IL-13Rα1 signaling pathway after IL-13 stimulation in IL-13Rα1 up-regulated and down-regulated ana-1 cells

**Figure 6:**
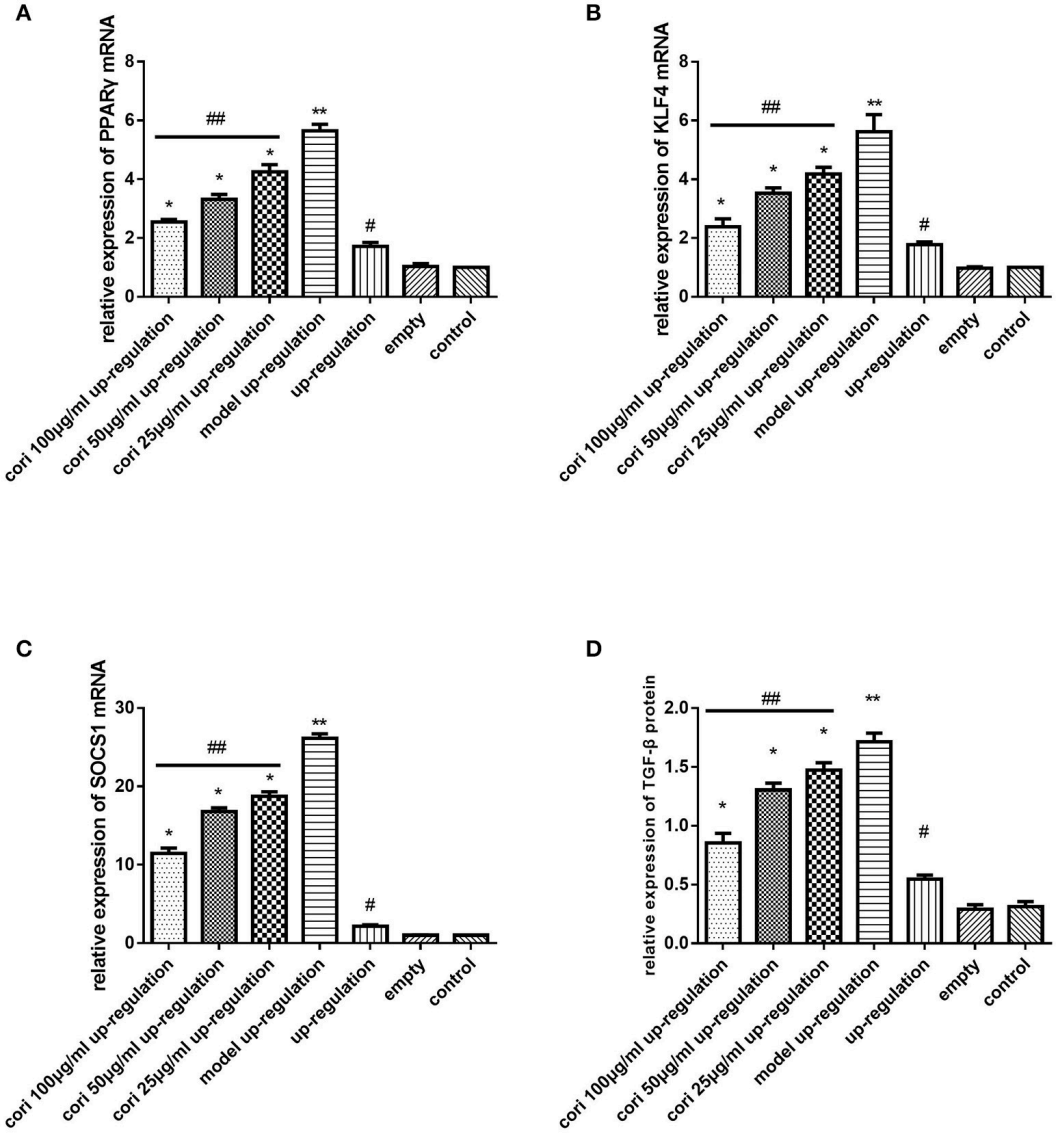
Effect of Corilagin on mRNA expression of PPARγ, KLF4, SOCS1, IL-13Rα1, STAT6 and protein expression of TGF-β after IL-13 stimulation in IL-13Rα1 up-regulated Ana-1 cells. Ana-1 cells was infected with IL-13Rα1 lentivirus or empty vector for 96 h except control group. Subsequently, the corilagin groups and model up-regulation group were administrated with rIL-13 for 24 h. Then the corilagin groups were treated with corilagin of different concentrations. Real-time qPCR and ELISA were performed. The dose-dependent anti-fibrogenesis effect of corilagin still persisted when IL-13Rα1 was up-regulated. **(A)** The PPARγ mRNA expression levels detected by real-time qPCR. ^*^p < 0.01 compared with the model up-regulation group (*n* = 3, student's *t*-test); ^**^p < 0.01 compared with the up-regulation group (*n* = 3, student's *t*-test); #*p* < 0.01 compared with the empty and control group (*n* = 3, student's *t*-test); ## *p* < 0.05 determined by one-way ANOVA and significant differences from the respective values determined by S-N-K method (*n* = 3). **(B)** The KLF4 mRNA expression levels detected by real-time qPCR. ^*^p < 0.01 compared with the model up-regulation group (*n* = 3, student's *t*-test); ^**^p < 0.01 compared with the up-regulation group (*n* = 3, student's *t*-test); #*p* < 0.01 compared with the empty and control group (*n* = 3, student's *t*-test); ##*p* < 0.05 determined by one-way ANOVA and significant differences from the respective values determined by S-N-K method (*n* = 3). **(C)** The SOCS1 mRNA expression levels detected by real-time qPCR. ^*^p < 0.01 compared with the model up-regulation group (*n* = 3, student's *t*-test); ^**^p < 0.01 compared with the up-regulation group (*n* = 3, student's *t*-test); #*p* < 0.01 compared with the empty and control group (*n* = 3, student's *t*-test); ##*p* < 0.05 determined by one-way ANOVA and significant differences from the respective values determined by S-N-K method (*n* = 3). **(D)** Effect of Corilagin on TGF-β protein expression detected by ELISA. ^*^p < 0.01 compared with the model up-regulation group (*n* = 3, student's *t*-test); ^**^p < 0.01 compared with the up-regulation group (*n* = 3, student's *t*-test); #*p* < 0.01 compared with the empty and control group (*n* = 3, student's *t*-test); ##*p* < 0.05 determined by one-way ANOVA and significant differences from the respective values determined by S-N-K method (*n* = 3). Data are shown with mean with SD.

**Figure 7:**
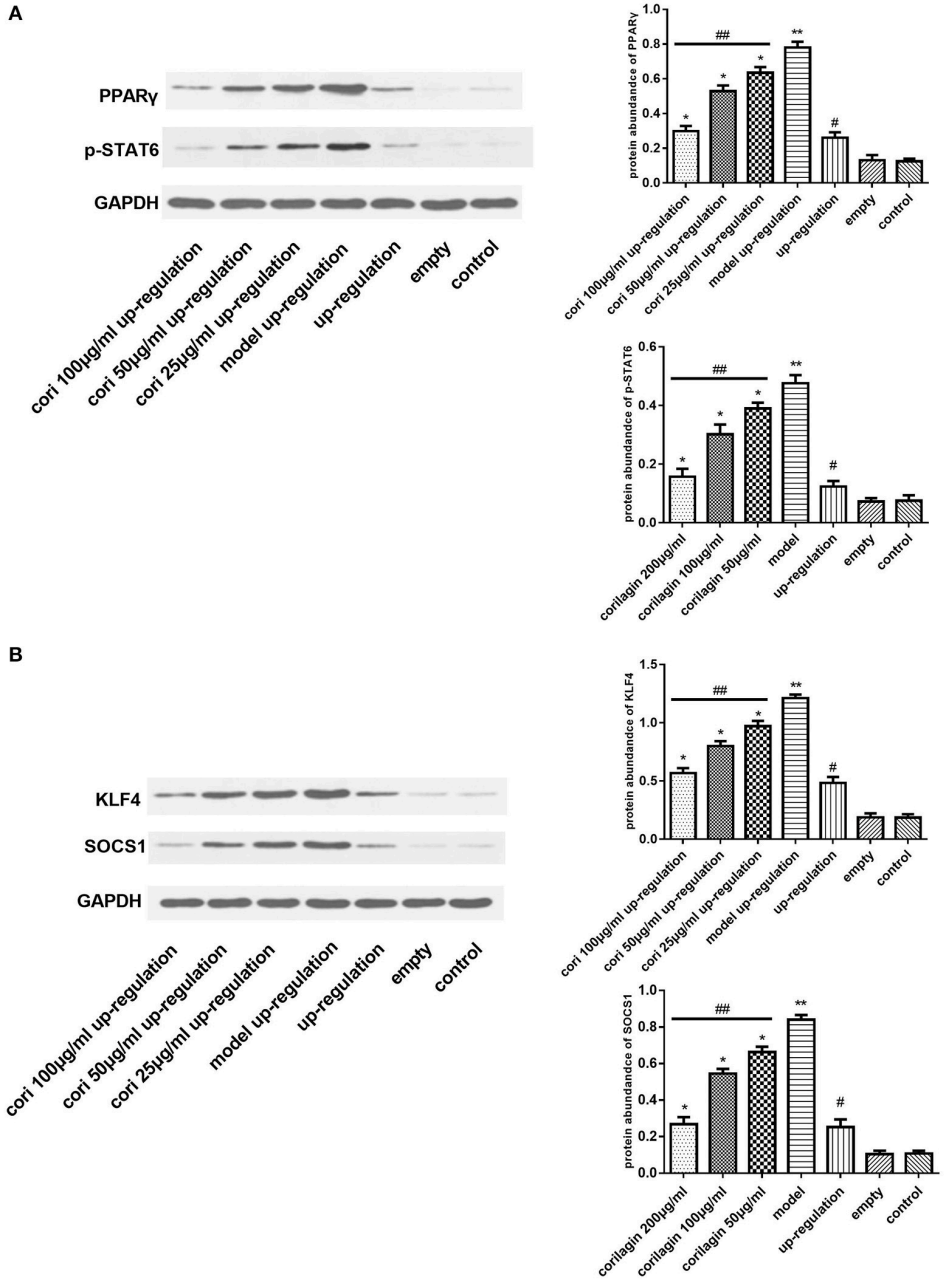
Effect of Corilagin on protein abundance of PPARγ, KLF4, SOCS1, and p-STAT6 after IL-13 stimulation in IL-13Rα1 up-regulated Ana-1 cells. Ana-1 cells was infected with IL-13Rα1 lentivirus or empty vector for 96 h except control group. Subsequently, the corilagin groups and model up-regulation group were administrated with rIL-13 for 24 h. Then the corilagin groups were treated with corilagin of different concentrations. Western blot assays were performed. The dose-dependent anti-fibrogenesis effect of corilagin still persisted when IL-13Rα1 was up-regulated. **(A)** The PPARγ and p-STAT6 protein abundance detected by western blot assay. ^*^p < 0.01 compared with the model up-regulation group (*n* = 3, student's *t*-test); ^**^p < 0.01 compared with the up-regulation group (*n* = 3, student's *t*-test); #*p* < 0.01 compared with the empty and control group (*n* = 3, student's *t*-test); ##*p* < 0.05 determined by one-way ANOVA and significant differences from the respective values determined by S-N-K method (*n* = 3). **(B)** The KLF4 and SOCS1 protein abundance detected by western blot assay. ^*^p < 0.01 compared with the model up-regulation group (*n* = 3, student's *t*-test); ^**^p < 0.01 compared with the up-regulation group (*n* = 3, student's *t*-test); #*p* < 0.01 compared with the empty and control group (*n* = 3, student's *t*-test); ##*p* < 0.05 determined by one-way ANOVA and significant differences from the respective values determined by S-N-K method (*n* = 3). Data are shown with mean with SD.

**Figure 8:**
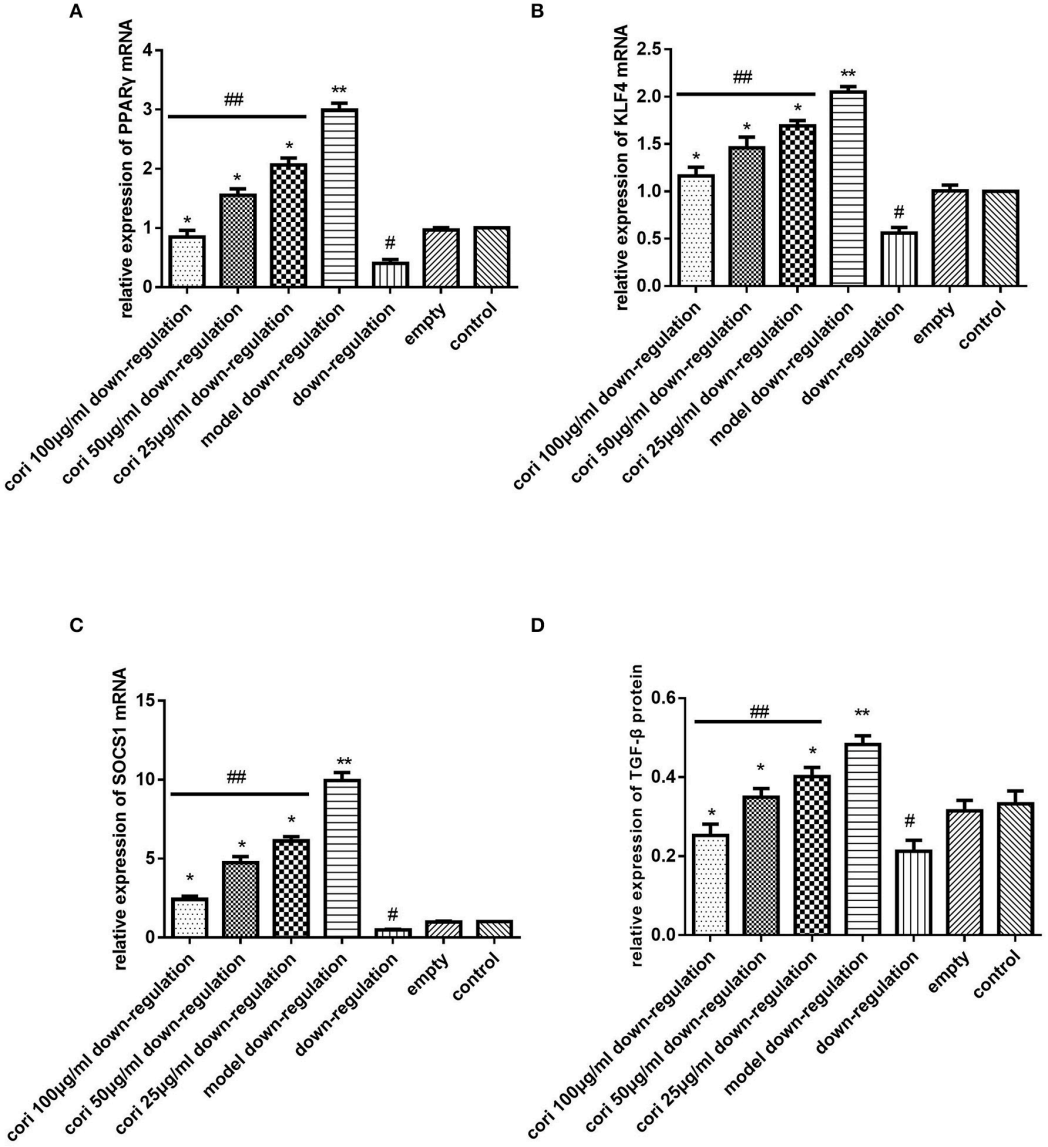
Effect of Corilagin on mRNA expression of PPARγ, KLF4, SOCS1, IL-13Rα1, STAT6, and protein expression of TGF-β after IL-13 stimulation in IL-13Rα1 down-regulated Ana-1 cells. Ana-1 cells was infected with IL-13Rα1 siRNA or empty vector for 48 h except control group. Subsequently, the corilagin groups and model down-regulation group were administrated with rIL-13 for 24 h. Then the corilagin groups were treated with corilagin of different concentrations. Real-time qPCR and ELISA were performed. The dose-dependent anti-fibrogenesis effect of corilagin still persisted when IL-13Rα1 was down-regulated. **(A)** The PPARγ mRNA expression levels detected by real-time qPCR. ^*^p < 0.01 compared with the model down-regulation group (*n* = 3, student's *t*-test); ^**^p < 0.01 compared with the down-regulation group (*n* = 3, student's *t*-test); #*p* < 0.01 compared with the empty and control group (*n* = 3, student's *t*-test); ##*p* < 0.05 determined by one-way ANOVA and significant differences from the respective values determined by S-N-K method (*n* = 3). **(B)** The KLF4 mRNA expression levels detected by real-time qPCR. ^*^p < 0.01 compared with the model down-regulation group (*n* = 3, student's *t*-test); ^**^p < 0.01 compared with the down-regulation group (*n* = 3, student's *t*-test); #*p* < 0.01 compared with the empty and control group (*n* = 3, student's *t*-test); ##*p* < 0.05 determined by one-way ANOVA and significant differences from the respective values determined by S-N-K method (*n* = 3). **(C)** The SOCS1 mRNA expression levels detected by real-time qPCR. ^*^p < 0.01 compared with the model down-regulation group (*n* = 3, student's *t*-test); ^**^p < 0.01 compared with the down-regulation group (*n* = 3, student's *t*-test); #*p* < 0.01 compared with the empty and control group (*n* = 3, student's *t*-test); ##*p* < 0.05 determined by one-way ANOVA and significant differences from the respective values determined by S-N-K method (*n* = 3). **(D)** Effect of Corilagin on TGF-β protein expression detected by ELISA. ^*^p < 0.01 compared with the model down-regulation group (*n* = 3, student's *t*-test); ^**^p < 0.01 compared with the down-regulation group (*n* = 3, student's *t*-test); #*p* < 0.01 compared with the empty and control group (*n* = 3, student's *t*-test); ##*p* < 0.05 determined by one-way ANOVA and significant differences from the respective values determined by S-N-K method (*n* = 3). Data are shown with mean with SD.

**Figure 9:**
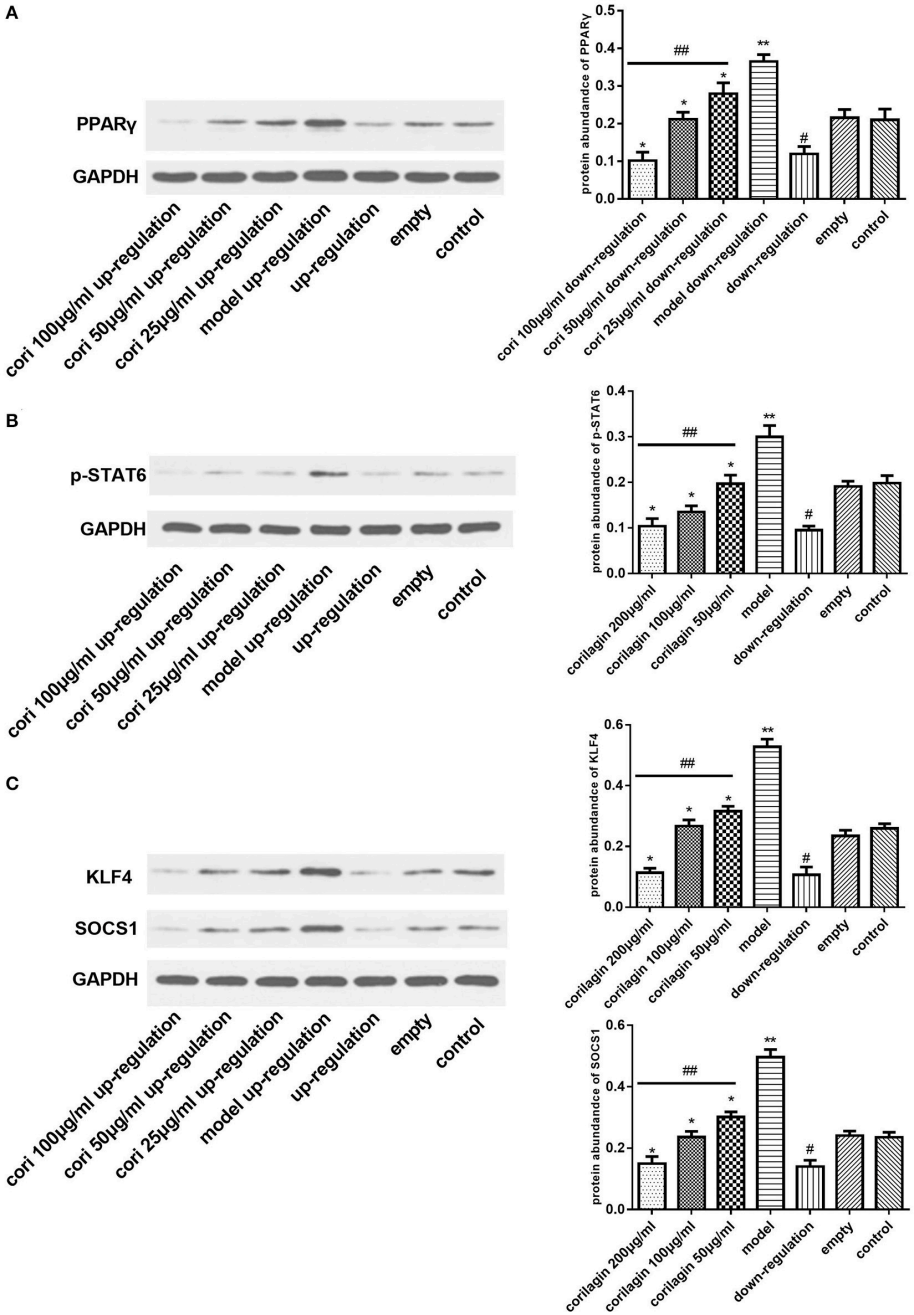
Effect of Corilagin on protein abundance of PPARγ, KLF4, SOCS1 and p-STAT6 after IL-13 stimulation in IL-13Rα1 down-regulated Ana-1 cells. Ana-1 cells was infected with IL-13Rα1 siRNA or empty vector for 48 h except control group. Subsequently, the corilagin groups and model down-regulation group were administrated with rIL-13 for 24 h. Then the corilagin groups were treated with corilagin of different concentrations. Western blot assays were performed. The dose-dependent anti-fibrogenesis effect of corilagin still persisted when IL-13Rα1 was down-regulated. **(A)** The PPARγ protein abundance detected by western blot assay. ^*^p < 0.01 compared with the model down-regulation group (*n* = 3, student's *t*-test); ^**^p < 0.01 compared with the down-regulation group (*n* = 3, student's *t*-test); #*p* < 0.01 compared with the empty and control group (*n* = 3, student's *t*-test); ##*p* < 0.05 determined by one-way ANOVA and significant differences from the respective values determined by S-N-K method (*n* = 3). **(B)** The p-STAT6 protein abundance detected by western blot assay. ^*^p < 0.01 compared with the model down-regulation group (*n* = 3, student's *t*-test); ^**^p < 0.01 compared with the down-regulation group (*n* = 3, student's *t*-test); #*p* < 0.01 compared with the empty and control group (*n* = 3, student's *t*-test); ##*p* < 0.05 determined by one-way ANOVA and significant differences from the respective values determined by S-N-K method (*n* = 3). **(C)** The KLF4 and SOCS1 protein abundance detected by western blot assay. ^*^p < 0.01 compared with the model down-regulation group (*n* = 3, student's *t*-test); ^**^p < 0.01 compared with the down-regulation group (*n* = 3, student's *t*-test); #*p* < 0.01 compared with the empty and control group (*n* = 3, student's *t*-test); ##*p* < 0.05 determined by one-way ANOVA and significant differences from the respective values determined by S-N-K method (*n* = 3). Data are shown with mean with SD.

Real-time qPCR was performed to examine the mRNA expression levels of PPARγ, KLF4 and SOCS1 in IL-13Rα1 up-regulated Ana-1 cells (Figures 6A–C) and down-regulated Ana-1 cells (Figures 8A–C). The TGF-β levels in the cell supernatant were determined by ELISA (Figures 6D, 8D). The abundance of PPARγ, KLF4, SOCS1 and p-STAT6 was determined by Western blot (Figures 7, 9). As illustrated, the levels of these molecules in the up- or down-regulation group correspondingly increased or decreased significantly compared with the control group (*p* < 0.01), indicating that IL-13Rα1 up- or down-regulation had a significant effect on the IL-13α1 signaling pathway. With IL-13 stimulation, the levels in the model up- or down-regulation group were increased significantly compared with those in the up- or down-regulation group (*p* < 0.01, Student's *t*-test, *n* = 3). The levels of these molecules with corilagin treatment of different concentrations were decreased compared with the model up- or down-regulation group (*p* < 0.01, Student's *t*-test, *n* = 3), and the inhibitory effects increased with the increasing concentration of corilagin (*p* < 0.05, one-way ANOVA, *post-hoc*: S-N-K method, *n* = 3), which revealed the dose-dependent inhibitory effect of corilagin on IL-13Rα1 signaling pathway still persisted when IL-13Rα1 was up- or down-regulated. The levels of these molecules in the empty-vector group showed no significant differences compared with the control group (*p* > 0.05, Student's *t*-test, *n* = 3).

### Effect of corilagin on IL-13Rα1 signaling pathway after praziquantel administration in schistosomiasis C57BL/6 mice

**Figure 10:**
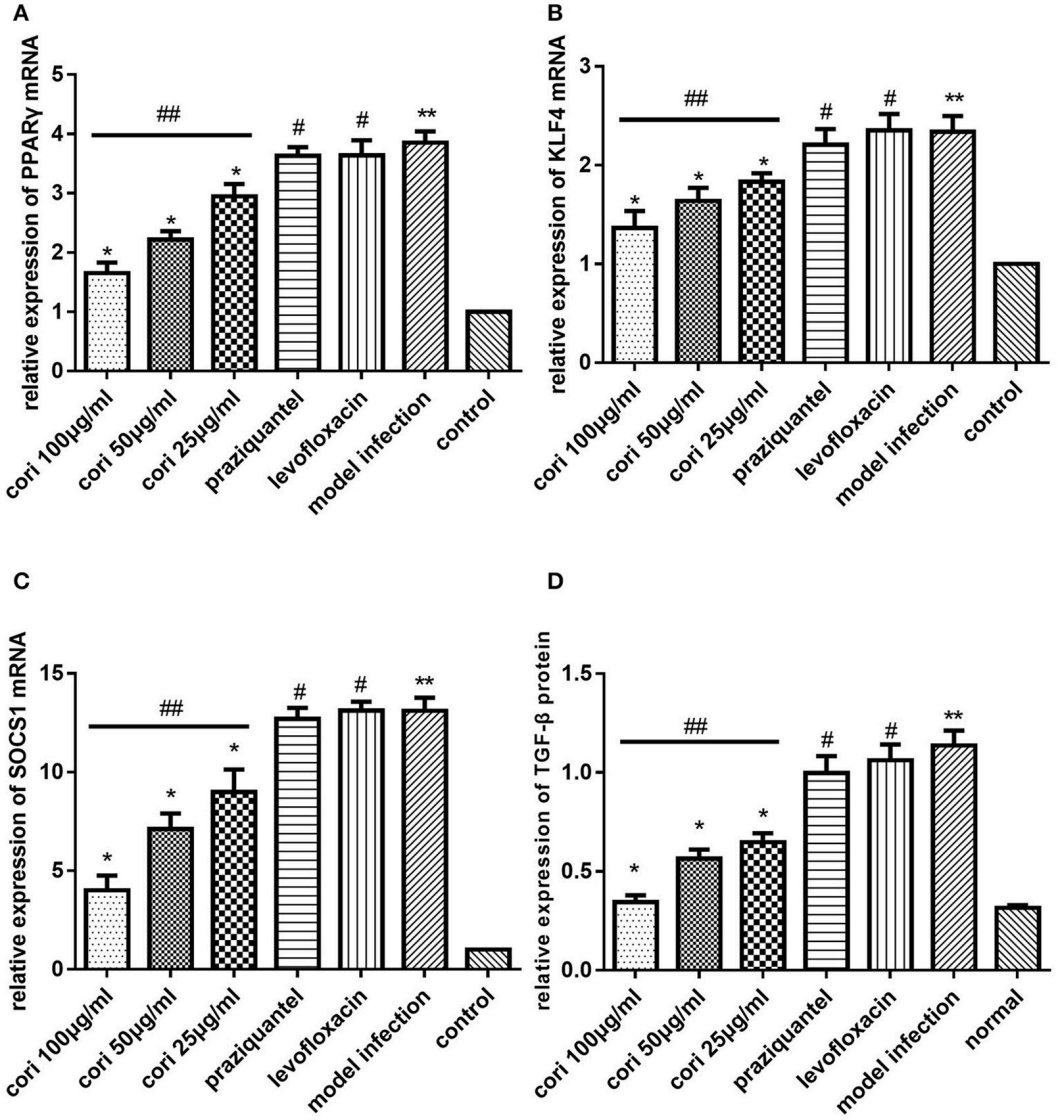
Effect of Corilagin on mRNA expression of PPARγ, KLF4, SOCS1, and protein expression of TGF-β in animal model. Twenty-eight days after the mice were infected with cercaria, 5-day praziquantel treatment was administrated to kill the adult schistosomes. Then the corilagin groups, praziquantel group and levofloxacin group were administrated with different drugs respectively for 21 days. Real-time qPCR and ELISA were performed. Corilagin showed a significant dose-dependent inhibitory effect on IL-13Rα1 signaling pathway *in vivo*. However, the praziquantel treatment had little effect. **(A)** The PPARγ mRNA expression levels detected by real-time qPCR. ^*^p < 0.01 compared with the model infection group (*n* = 6, student's *t*-test); ^**^p < 0.01 compared with the control group (*n* = 6, student's *t*-test); #*p* < 0.01 compared with the control group (*n* = 6, student's *t*-test); ##*p* < 0.05 determined by one-way ANOVA and significant differences from the respective values determined by S-N-K method (*n* = 6). **(B)** The KLF4 mRNA expression levels detected by real-time qPCR. ^*^p < 0.01 compared with the model infection group (*n* = 6, student's *t*-test); ^**^p < 0.01 compared with the control group (*n* = 6, student's *t*-test); #*p* < 0.01 compared with the control group (*n* = 6, student's *t*-test); ##*p* < 0.05 determined by one-way ANOVA and significant differences from the respective values determined by S-N-K method (*n* = 6). **(C)** The SOCS1 mRNA expression levels detected by real-time qPCR. ^*^p < 0.01 compared with the model infection group (*n* = 6, student's *t*-test); ^**^p < 0.01 compared with the control group (*n* = 6, student's *t*-test); #*p* < 0.01 compared with the control group (*n* = 6, student's *t*-test); ##*p* < 0.05 determined by one-way ANOVA and significant differences from the respective values determined by S-N-K method (*n* = 6). **(D)** Effect of Corilagin on TGF-β protein expression detected by ELISA. ^*^p < 0.01 compared with the model infection group (*n* = 6, student's *t*-test); ^**^p < 0.01 compared with the control group (*n* = 6, student's *t*-test); #*p* < 0.01 compared with the control group (*n* = 6, student's *t*-test); ##*p* < 0.05 determined by one-way ANOVA and significant differences from the respective values determined by S-N-K method (*n* = 6). Data are shown with mean with SD.

**Figure 11:**
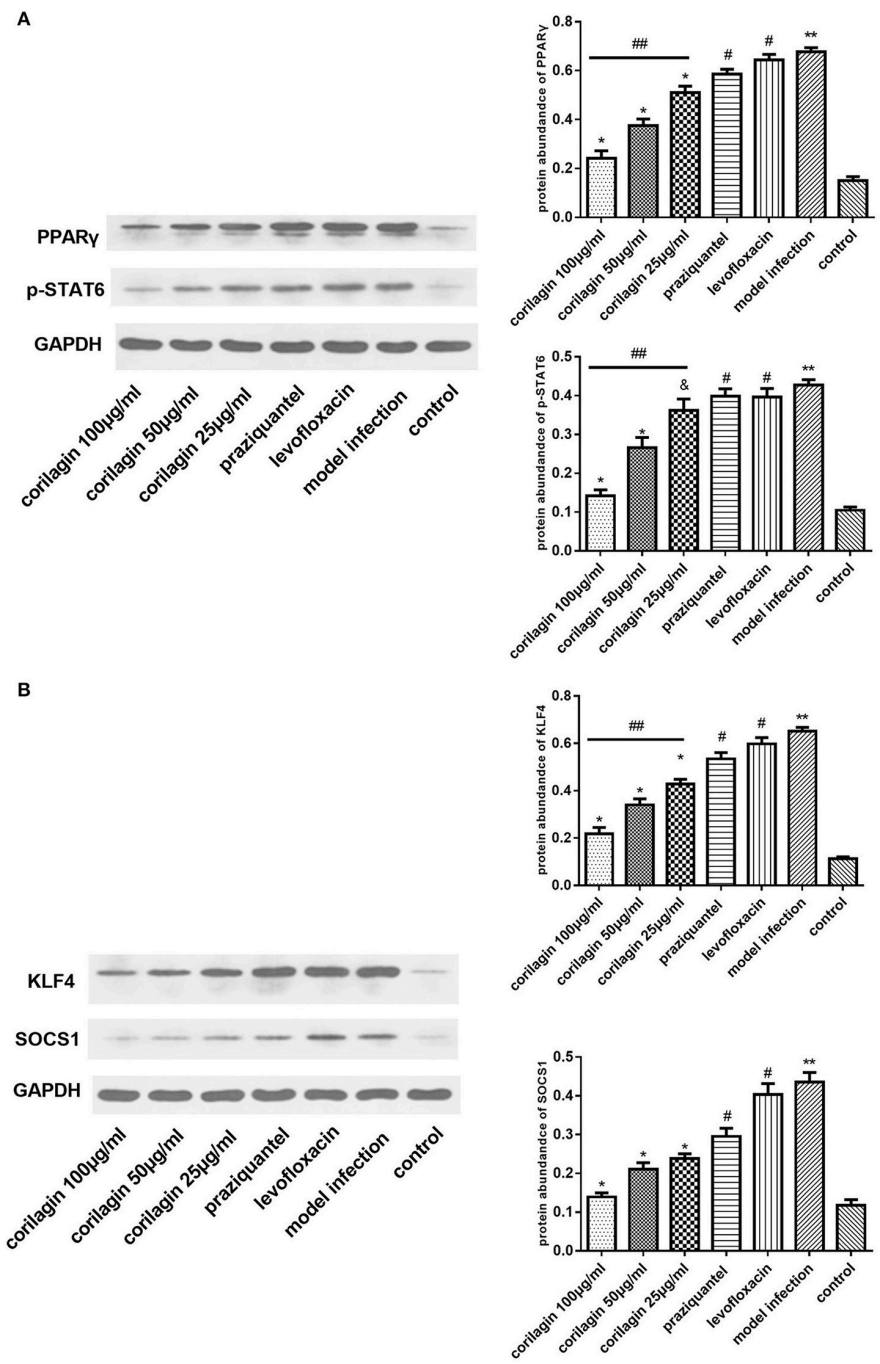
Effect of Corilagin on protein abundance of PPARγ, KLF4, SOCS1, and p-STAT6 in animal model. After the mice model was established as we stated, western blot assays were performed. Corilagin showed a significant dose-dependent inhibitory effect on IL-13Rα1 signaling pathway *in vivo*. However, the praziquantel treatment had little effect. **(A)** The PPARγ and p-STAT6 protein abundance detected by western blot assay. ^*^p < 0.01 compared with the model infection group (*n* = 6, student's *t*-test); &*p* < 0.05 compared with the model infection group (*n* = 6, student's *t*-test); ^**^p < 0.01 compared with the control group (*n* = 6, student's *t*-test); #*p* < 0.01 compared with the control group (*n* = 6, student's *t*-test); ##*p* < 0.05 determined by one-way ANOVA and significant differences from the respective values determined by S-N-K method (*n* = 6). **(B)** The KLF4 and SOCS1 protein abundance detected by western blot assay. ^*^p < 0.01 compared with the model infection group (*n* = 6, student's *t*-test); ^**^p < 0.01 compared with the control group (*n* = 6, student's *t*-test); #*p* < 0.01 compared with the control group (*n* = 6, student's *t*-test); ##*p* < 0.05 determined by one-way ANOVA and significant differences from the respective values determined by S-N-K method (*n* = 6). Data are shown with mean with SD.

In the animal model, we quantified the mRNA expression of PPARγ, KLF4 and SOCS1 in liver tissue using real-time qPCR (Figures 10A–C). The level of TGF-β in mice serum was measured by ELISA (Figure 10D). The abundance of PPARγ, KLF4, SOCS1 and p-STAT6 in liver tissue was measured by Western blot (Figure 11). As shown, the levels of these molecules in the model infection group were increased compared with the control group (*p* < 0.01, Student's *t*-test, *n* = 6). The levels of these molecules with corilagin treatment of different concentrations were decreased compared with the model infection group (*p* < 0.01 or *p* < 0.05, Student's *t*-test, *n* = 6), and the inhibitory effects increased with increasing concentrations of corilagin (*p* < 0.05, one-way ANOVA, *post-hoc*: S-N-K method, *n* = 6), which confirmed the dose-dependent inhibitory effect of corilagin on IL-13Rα1 signaling pathway *in vivo*. The levels of these molecules in the praziquantel group (positive control) and levofloxacin group (negative control) were increased compared with the control group (*p* < 0.01, Student's *t*-test, *n* = 6) and showed no significant differences compared with the model infection group (*p* > 0.05, Student's *t*-test, *n* = 6), which indicated praziquantel had little effect on IL-13Rα1 signaling pathway.

### Effect of corilagin on pathological changes of schistosomiasis liver fibrosis in the mouse liver

**Figure 12:**
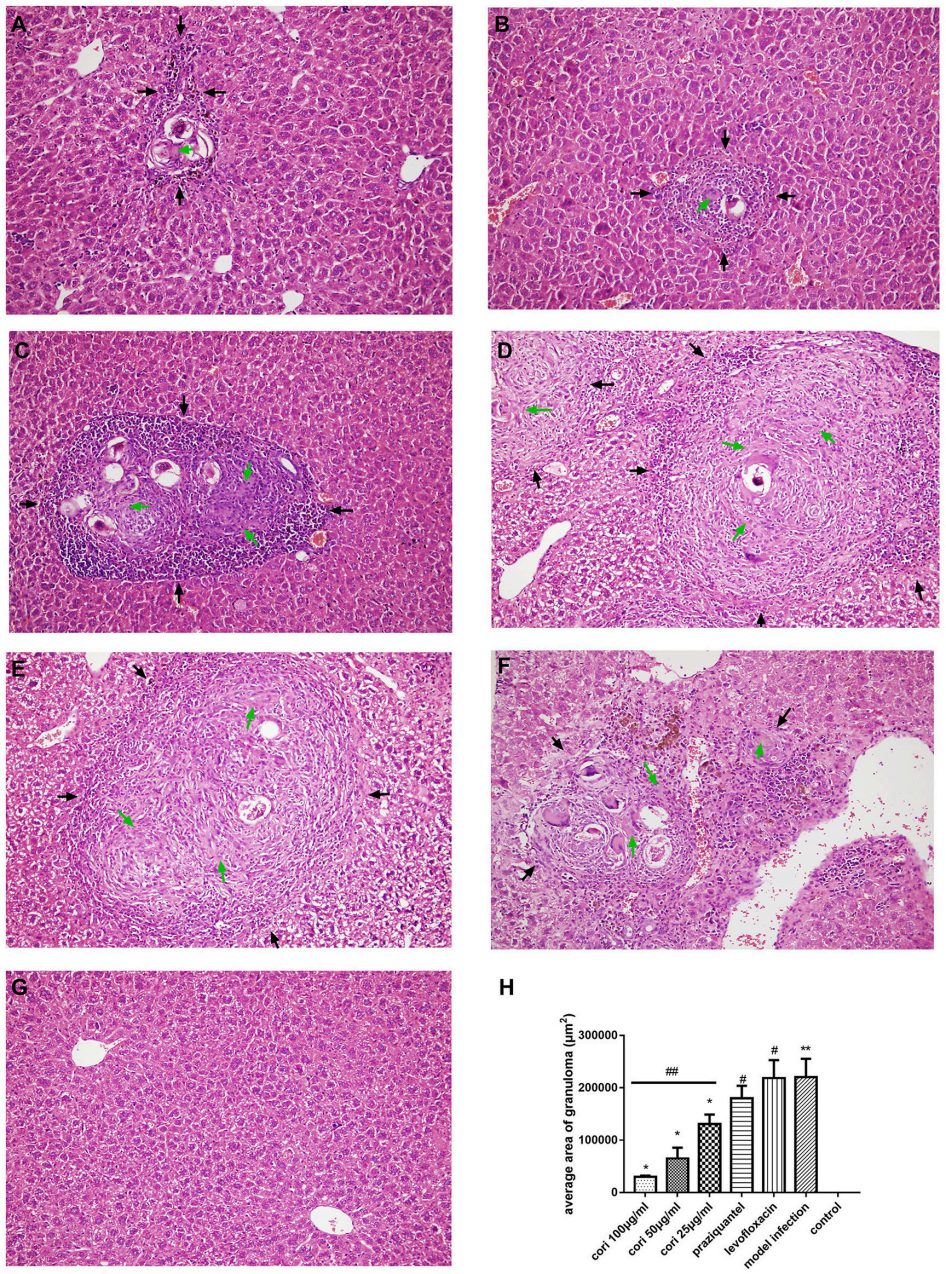
Effect of Corilagin on schistosomiasis fibrosis in animal model examined by HE staining in 200× magnification. After the mice model was established, HE staining was performed. In each figure, the black arrows indicate the area of fibrotic nodules and the green arrows indicate the fibrin which was stained pink in HE staining. Corilagin treatment significantly reduced the granuloma area *in vivo*. However, the praziquantel treatment had little effect. **(A)** High-concentration corilagin group; **(B)** medium-concentration corilagin group; **(C)** low-concentration corilagin group; **(D)** praziquantel group; **(E)** levofloxacin group; **(F)** model infection group; **(G)** control group; **(H)** average area of granuloma. ^*^p < 0.01 compared with the model infection group (*n* = 6, student's *t*-test); ^**^p < 0.05 compared with the control group (*n* = 6, student's *t*-test); #*p* < 0.01 compared with the control group (*n* = 6, student's *t*-test); ##*p* < 0.05 determined by one-way ANOVA and significant differences from the respective values determined by S-N-K method (*n* = 6). Data are shown with mean with SD.

**Figure 13:**
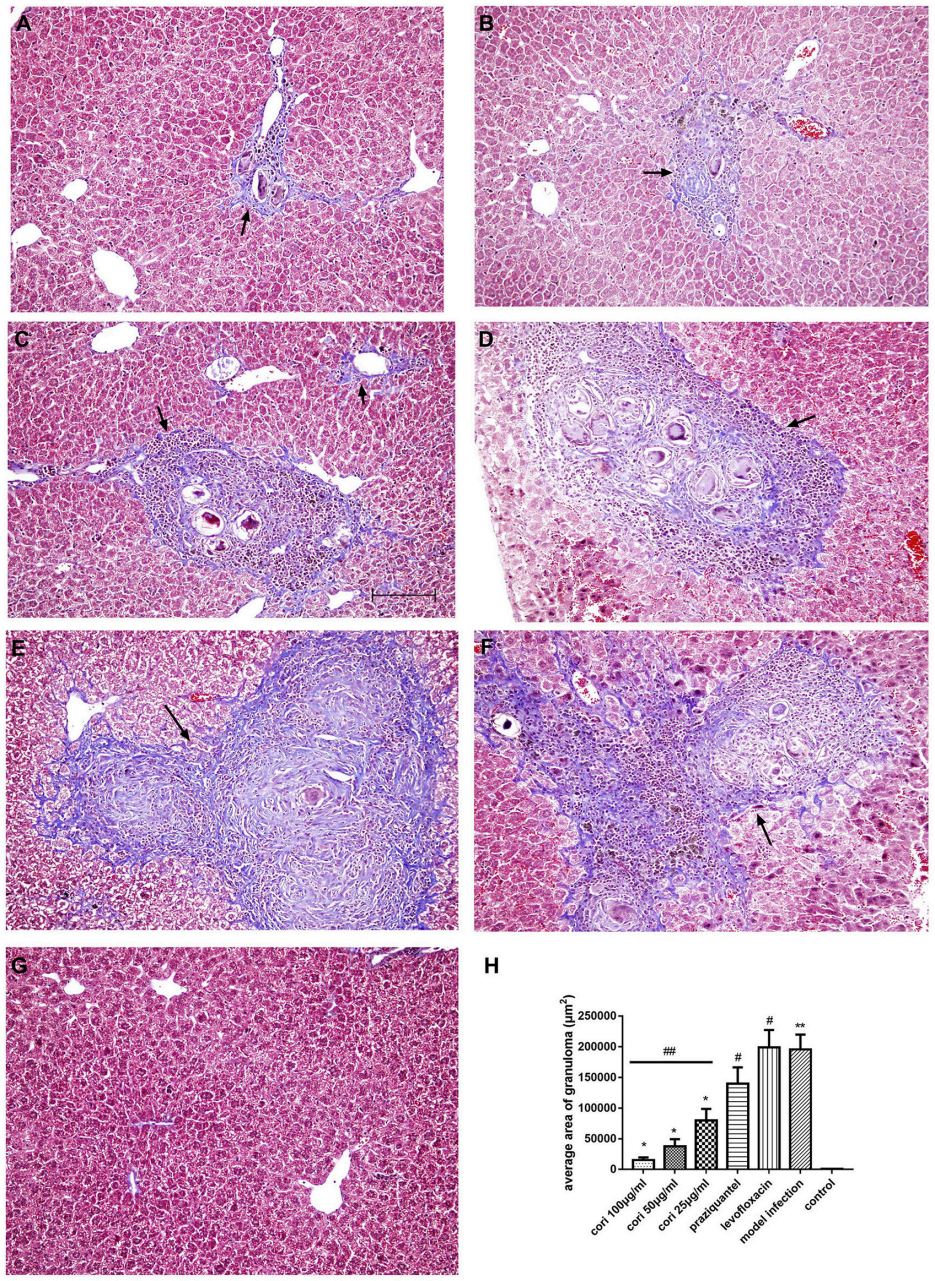
Effect of Corilagin on schistosomiasis fibrosis in animal model examined by Masson staining in 200× magnification. After the mice model was established, HE staining was performed. Black arrows indicate the area of fibrosis which was stained purple in Masson staining. Corilagin treatment significantly reduced the fibrosis area *in vivo*. However, the praziquantel treatment had little effect. **(A)** high-concentration corilagin group; **(B)** medium-concentration corilagin group; **(C)** low-concentration corilagin group; **(D)** praziquantel group; **(E)** levofloxacin group; **(F)** model infection group; **(G)** control group; **(H)** average area of fibrosis. ^*^p < 0.05 compared with the model infection group (*n* = 6, student's *t*-test); ^**^p < 0.01 compared with the control group (*n* = 6, student's *t*-test); #*p* < 0.01 compared with the control group (*n* = 6, student's *t*-test); ##*p* < 0.05 determined by one-way ANOVA and significant differences from the respective values determined by S-N-K method (*n* = 6). Data are shown with mean with SD.

**Figure 14:**
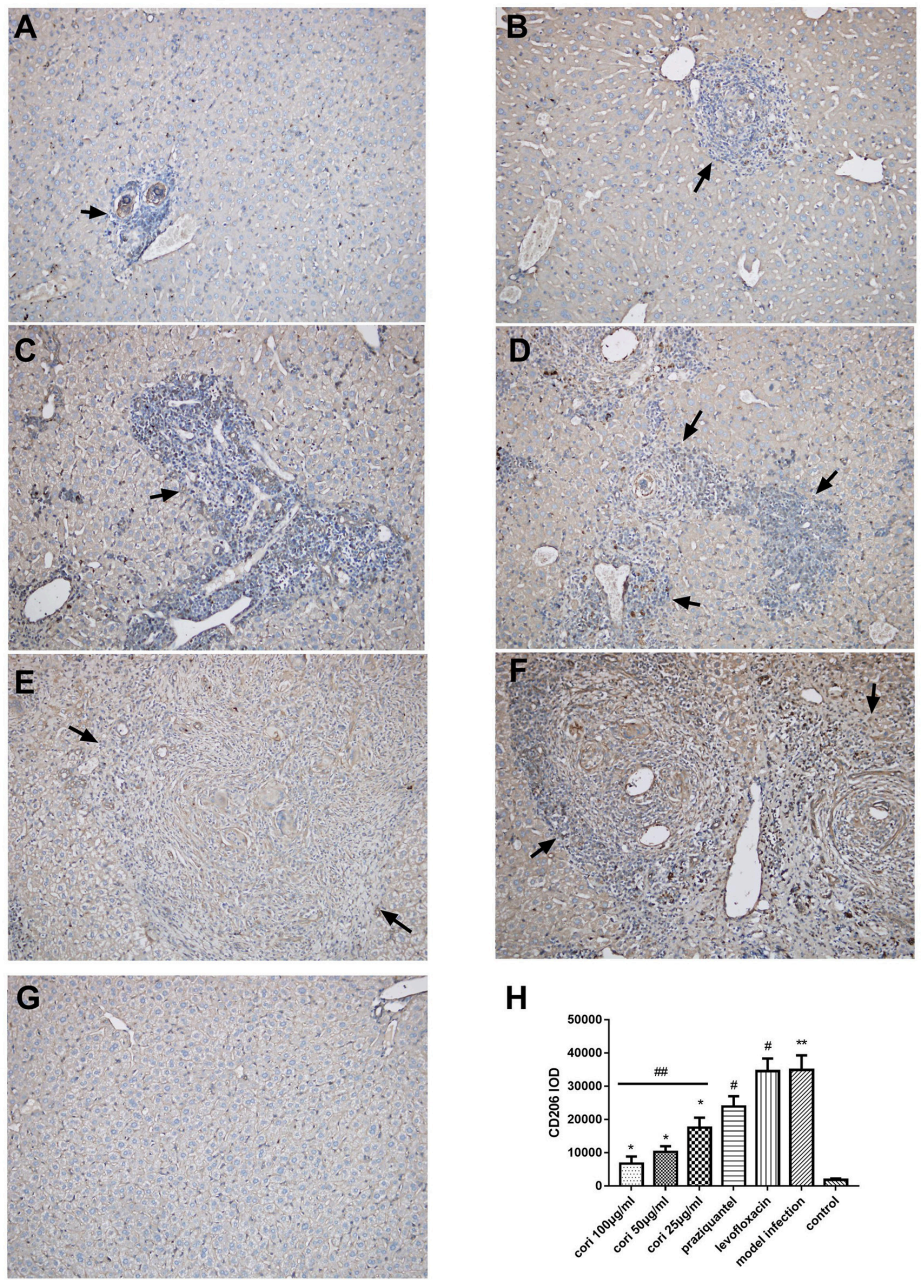
Effect of Corilagin on the distribution of M2 macrophages in animal model examined by immunohistochemistry (200×). After the mice model was established, immunohistochemistry for CD206 was performed. Black arrows indicate the distribution of M2 macrophage. Corilagin treatment significantly reduced the distribution of M2 macrophages *in vivo*. However, the praziquantel treatment had little effect. **(A)** High-concentration corilagin group; **(B)** medium-concentration corilagin group; **(C)** low-concentration corilagin group; **(D)** praziquantel group; **(E)** levofloxacin group; **(F)** model infection group; **(G)** control group; **(H)** CD206 IOD analysis. ^*^p < 0.05 compared with the model infection group (*n* = 6, student's *t*-test); ^**^p < 0.01 compared with the control group (*n* = 6, student's *t*-test); #*p* < 0.01 compared with the control group (*n* = 6, student's *t*-test); ##*p* < 0.05 determined by one-way ANOVA and significant differences from the respective values determined by S-N-K method (*n* = 6). Data are shown with mean with SD.

The effect of corilagin on pathological changes of schistosomiasis liver fibrosis in mice liver was observed with HE, Masson staining and immunohistochemistry. According to the results, all the liver tissue of the infected mice showed varying degrees of fibrosiss, which indicated the model establishment was successful. As illustrated in Figure 12, significant fibrosis and granuloma were observed in the model infection, praziquantel and levofloxacin groups, and the area of granuloma was remarkably larger than that in the control group (*p* < 0.01, Student's *t*-test, *n* = 6). The area of granuloma with corilagin treatment of different concentrations compared was significantly smaller with the model infection groups (*p* < 0.05, Student's *t*-test, *n* = 6), and the area decreased with increasing concentrations of corilagin (*p* < 0.05, one-way ANOVA, *post-hoc*: S-N-K method, *n* = 6), which showed a dose-dependent anti-fibrogenesis effect. For pathological scores, apart from normal group, the inflammation score of high-concentration corilagin group and medium-concentration corilagin group was G2, while low-concentration corilagin group was G2-G3, and praziquantel group was G3. The inflammation score of levofloxacin group and model infection group was G3-G4. The fibrosis score of corilagin groups was S2 and praziquantel, levofloxacin group and model infection group was S3. As illustrated in Figure 13, and the area of fibrosis in the model infection, praziquantel and levofloxacin groups was remarkably larger than that in the control group (*p* < 0.01, Student's *t*-test, *n* = 6). The area of fibrosis with corilagin treatment of different concentrations compared was significantly smaller compared with the model infection groups (*p* < 0.05, Student's *t*-test, *n* = 6), and the area decreased with increasing concentrations of corilagin (*p* < 0.05, one-way ANOVA, *post-hoc*: S-N-K method, *n* = 6), which showed a dose-dependent anti-fibrogenesis effect. As illustrated in Figure 14, the levels of CD206 staining in the model infection, praziquantel and levofloxacin groups were significantly higher than that in the control group (*p* < 0.01, Student's *t*-test, *n* = 6). The levels of CD206 staining were significantly lower in the corilagin groups compared with the model infection groups (*p* < 0.05, Student's *t*-test, *n* = 6), and the area decreased with increasing concentrations of corilagin (*p* < 0.05, one-way ANOVA, *post-hoc*: S-N-K method, *n* = 6). The less area of fibrosis and distribution of M2 macrophages in corilagin treatment group indicated the anti-fibrogenic effect of corlagin *in vivo*.

## Discussion

Schistosomiasis hepatic fibrosis is a complex pathological process involving multiple cell-signaling pathways and cytokines. M2 macrophages play a critical role in the pathogenesis of this disease (He et al., 2016). M2 macrophages are produced upon the stimulation of IL-13 (Gordon, 2003), which is significantly up-regulated during schistosome infection. The expression of M2 macrophage markers such as Arg-1, Ym-1, and Fizz-1 were highly increased in schistosomiasis (Sandler et al., 2003). These markers induce L-arginine to be converted to proline, an essential amino acid for the production of collagen (Hesse et al., 2001). Apart from the pro-fibrotic cytokines produced by M2 macrophages, M2 macrophages are an important component of granuloma in schistosomiasis (Ragheb and Boros, 1989).

M2 polarization of macrophages is induced by the IL-13-mediated Th2 immune response and eventually leads to the secretion of pro-fibrotic cytokines. IL-13 binding to the IL-4Rα/IL-13Rα1 receptor complex triggers phosphorylation of STAT6, further promoting the expression of downstream pro-fibrotic molecules (Anthony et al., 2012). In this study, we aimed to explore the effects of corilagin in controlling egg-induced hepatic fibrosis after the adult schistosomes were killed and to detect the expression of IL-13α1 and the downstream M2 molecules, which suggested that the mechanism of suppressing egg-induced hepatic fibrosis was mediated by inhibiting the activation of IL-13α1 signaling pathway and M2 polarization of macrophages.

IL-13Rα1 is a central mediator of the Th2-biased response and schistosomiasis hepatic fibrosis (Beschin et al., 2013). Thus, we up- and down-regulated the expression of IL-13Rα1 in Ana-1 cells, attempting to evaluate the effect of corilagin in the case that the sensitivity of Ana-1 cells to IL-13 was changed. We found that the levels of IL-13Rα1 mRNAs showed no significant differences in the model group compared with the corilagin groups and the control group, which indicated corilagin had no significant effect on the amount of IL-13Rα1. Meanwhile, the expression levels of other signaling molecules in IL-13Rα1 pathway such as PPARγ, KLF4, SOCS1, p-STAT6 and TGF-β were significantly suppressed in the corilagin groups compared with the model group. It is known that IL-13Rα1 has a much lower affinity for IL-13 than IL-13Rα2, but the affinity is greatly enhanced when it dimerizes with IL-4Rα to form a receptor complex (Wang et al., 2011). Therefore, we speculate that the inhibitory effect of corilagin on the fibrosis induced by IL-13 is not achieved through altering the expression of IL-13Rα1. Instead, corilagin might reduce the affinity of IL-13Rα1 for IL-13 or interfere with the binding of IL-13 to IL-13Rα1. However, these assumptions remain to be confirmed by further experiments. In IL-13Rα1 up-/down-regulation experiments, it revealed that no matter how the levels of IL-13Rα1 changed, corilagin still showed a significant inhibitory effect on pro-fibrotic cytokines.

Besides IL-13Rα1, we measured the expression of other important molecules in IL-13Rα1 signaling pathway. As one of the key transcription factors in the IL-13α1 signaling pathway, STAT6 plays an important role in fibrosis in multiple organs (Edwards, 2015; Su et al., 2015). PPARγ, KLF4 and TGF-β are also the important pro-fibrotic downstream molecules in the IL-13α1 pathway. PPARγ is involved in IL-13 mediated fibrogenesis in multiple organs (Bou Saab et al., 2016; Yu et al., 2016) and in M2 polarization of macrophages (Assuncao et al., 2017). Several studies have demonstrated that inhibition of PPARγ can alleviate schistosomiasis hepatic fibrosis (Attia et al., 2013; Duan et al., 2014). KLF4 is a critical molecule in M2 macrophage polarization and can inhibit M1 macrophage polarization; both processes promote fibrosis (Sica and Mantovani, 2012; Ke et al., 2015). TGF-β can be induced by SEA and strongly stimulates the secretion of collagen (Baghy et al., 2012; Dooley and ten Dijke, 2012). We found that STAT6 phosphorylation, PPARγ, KLF4 and TGF-β were reduced by corilagin treatment. In cellular and animal experiments, the praziquantel group showed no significant differences compared to the model group, which indicated that praziquantel did not inhibit the IL-13 signaling pathway. Decreased expression of these pro-fibrotic molecules with corilagin administration suggests an inhibitory effect of corilagin on IL-13-induced M2 polarization of macrophages and fibrogenesis.

SOCS1 is an important negative-feedback regulator of STAT6 phosphorylation that competes with STAT6 for phosphorylation binding sites of JAK (Su et al., 2015; Kandhi et al., 2016). PPARγ and SOCS1 expression by macrophages increased with IL-4 stimulation (Su et al., 2015). The expression of SOCS1 increased notably following administration of IL-13, probably as a consequence of feedback from increased STAT6 phosphorylation. SOCS1 expression increases with increasing phosphorylation of STAT6, as a part of negative-feedback regulation of p-STAT6.

To confirm the inhibitory effect of corilagin on egg-induced fibrosis *in vivo*, we observed pathological changes in the mouse liver by examining tissue sections in animal experiments. HE staining was performed to observe the morphological characteristics of the cells and the area of granuloma, and Masson staining was performed to observe the degree of fibrosis in the area of deep staining. It is admitted that CD206 is a hallmark of M2 macrophage (Wollenberg et al., 2002; Aron-Wisnewsky et al., 2009; Biswas and Mantovani, 2010). Therefore, immunohistochemistry for CD206 was performed to observe the distribution of M2 macrophages. In HE and Masson staining of liver tissue sections, less fibrosis, granuloma and distribution of M2 macrophages were observed in the liver tissue of mice treated with corilagin intervention than in the model infection, praziquantel or levofloxacin groups. We used praziquantel treatment as a positive control and levofloxacin treatment as a negative control. At 28 days post-infection, all the infected mice were administered praziquantel for 5 days to kill the adult schistosomes. Though the adult schistosomes were killed by praziquantel, the *Schistosoma* eggs continued to secrete SEA, inducing fibrogenesis. As observed in tissue sections, fibrosis in the praziquantel group and levofloxacin group was much more severe than in the control group and showed no significant difference compared to the model infection group, indicating that the praziquantel has little effect on the progression of fibrogenesis. However, fibrosis was suppressed significantly in the corilagin groups compared with the model infection, praziquantel and levofloxacin groups, demonstrating that corilagin inhibited egg-induced fibrosis more than praziquantel did *in vivo*.

In this study, we demonstrated the inhibitory effect of corilagin on M2 macrophages and schistosome-egg-induced hepatic fibrosis via IL-13Rα1 signaling pathway *in vitro* and *in vivo*, especially after the adult schistosomes were killed. Based on the cellular experiment including up- and down-regulation of IL-13Rα1, we speculated that the results might be attributed to corilagin's interference in the dimerization of IL-13Rα1 and IL-4Rα and (or) the binding of IL-13 to IL-13Rα1, leading to decreased affinity of the receptor for IL-13. However, more direct evidence is needed, and we will continue to focus on this aspect. We planned to clarify the further mechanism of corilagin on egg-induced hepatic fibrosis with IL-13 knock-out mice *in vivo*. Moreover, in view of the crucial role of STAT6 in signal transduction, we will continue to examine this molecule through studies of up- and down-regulation to explore its influence on downstream molecules such as PPARγ, KLF4, SOCS1 and TGF-β, and whether STAT6 contributes the anti-fibrogenic effect of corilagin. As currently the effective drug of egg-induced hepatic fibrosis is still relatively lacking, researchers are always trying to find a new drug to treat this disease. In our animal experiments, corilagin more effectively suppressed fibrosis after the adult schistosomes were killed than did praziquantel, which gave us more confidence in this compound that it might provide an extra choice for the patients suffering from this disease and relieve their pain. Based on our experience on the medical effects of natural ingredients (Dang et al., 2017), we believe that corilagin has potential to control schistosome egg-induced hepatic fibrosis in human, bringing new hope to schistosomiasis patients and their family.

## Author contributions

Conceived and designed the experiments: LZ. Performed the experiments and analyzed the data: YL, YC, and YD. Contributed reagents/materials/analysis tools and wrote the paper: YL, YC, YD, and LZ. Critical revision of the paper for important intellectual content: YL, YC, YD, YW, ZS, QM, YJW, JZ, LL, QL, and LZ. All authors agree with final approval of the version for submission.

### Conflict of interest statement

The authors declare that the research was conducted in the absence of any commercial or financial relationships that could be construed as a potential conflict of interest.

## Abbreviations

ANOVA: Analysis of variance
ELISA: Enzyme-Linked Immunosorbent Assay
FBS: fetal bovine serum
HE staining: Haematoxylin and eosin staining
HSC: Hepatic stellate cell
HSV1: Herpes simplex virus 1
IL-4Rα: Interleukin-4 receptor alpha
IL-13: Interleukin-13
IL-13Rα1: Interleukin-13 receptor alpha 1
KLF4: Krüppel-like factor4
MOI: Multiplicity of infection
mRNA: Messenger ribonucleic acid
NF-κB: Nuclear factor kappa-light-chain-enhancer of activated B cells
PBS: Phosphate buffer saline
PPARγ: Peroxisome proliferator-activated receptor gamma
p-STAT6: Phosphorylated signal transducer and activator of transcription 6
qPCR: Quantitative polymerase chain reaction
RPMI medium: Roswell Park Memorial Institute medium
SD: Standard deviation
SEA: Soluble egg antigen
siRNA: Small interfering ribonucleic acid
SOCS1: Suppressor of cytokine signaling 1
STAT6: Signal transducer and activator of transcription 6
TGF-β: Transforming growth factor beta
TNF-α: tumor necrosis factor alpha
WB: Western blot.
