# Supplementary material for: Corilagin Counteracts IL-13Rα1 Signaling Pathway in Macrophages to Mitigate Schistosome Egg-Induced Hepatic Fibrosis
Source: Front Cell Infect Microbiol. 2017 Oct 18;7:443. doi: 10.3389/fcimb.2017.00443 (PMC5651236; doi:10.3389/fcimb.2017.00443)
Supplement: Supplementary file 1 [file DataSheet1.PDF]

Supplement Figure 1: Standard cruves

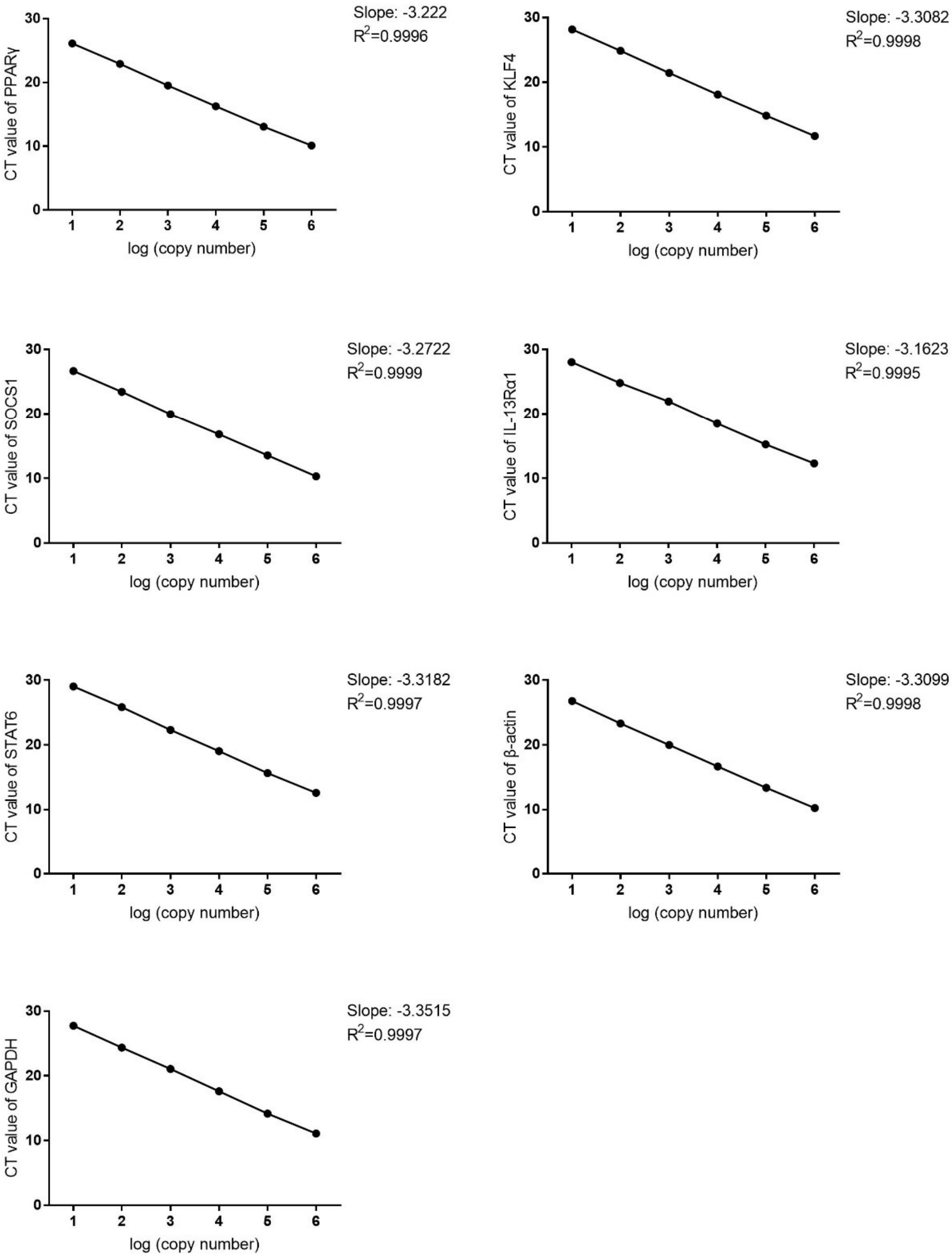

Supplement Figure 2: Amplification curves

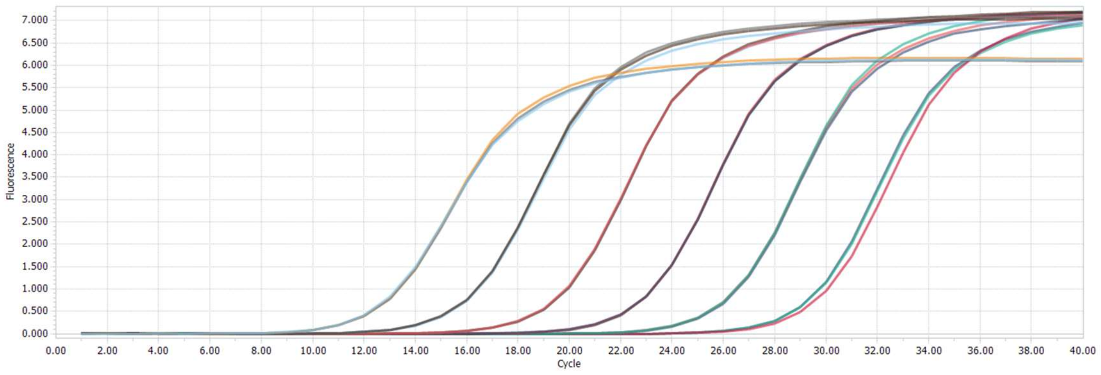

GAPDH

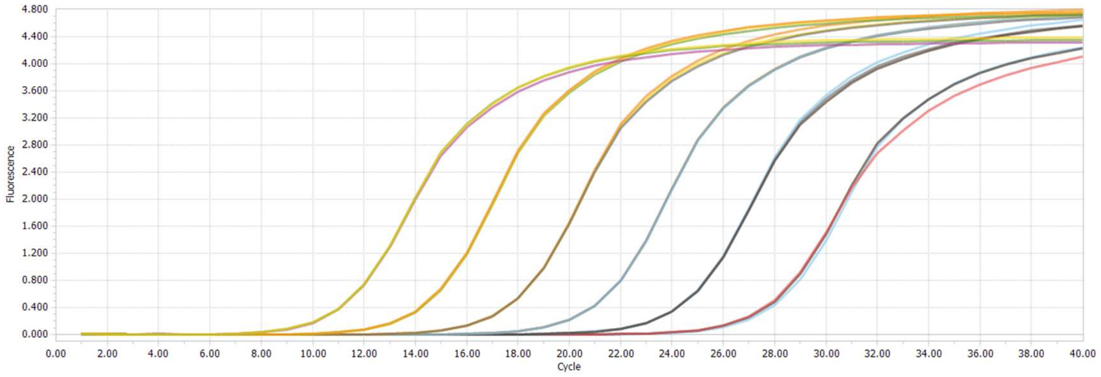

β-actin

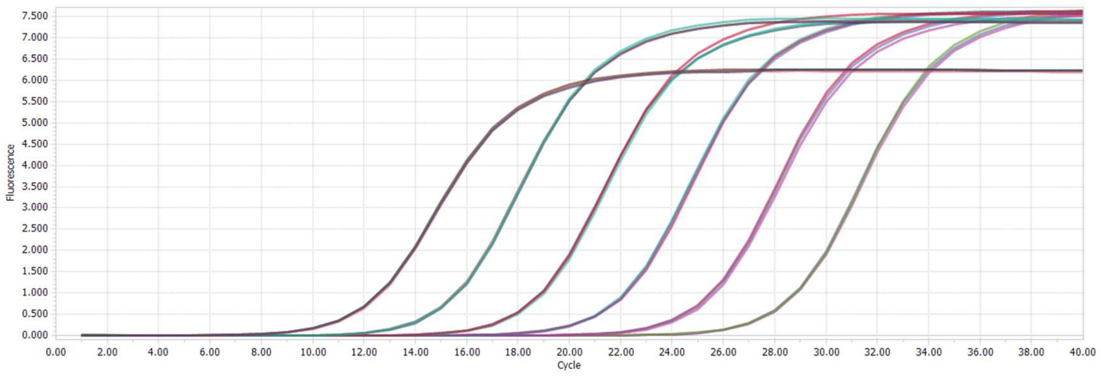

IL-13Rα1

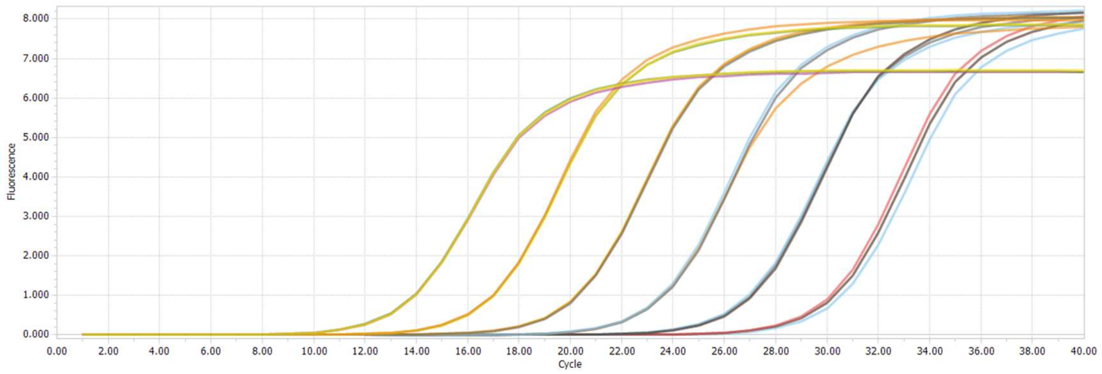

KLF4

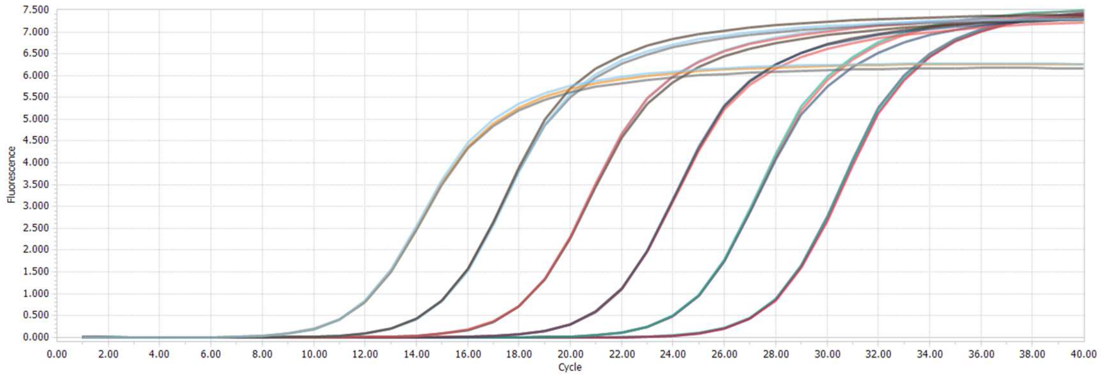

PPARγ

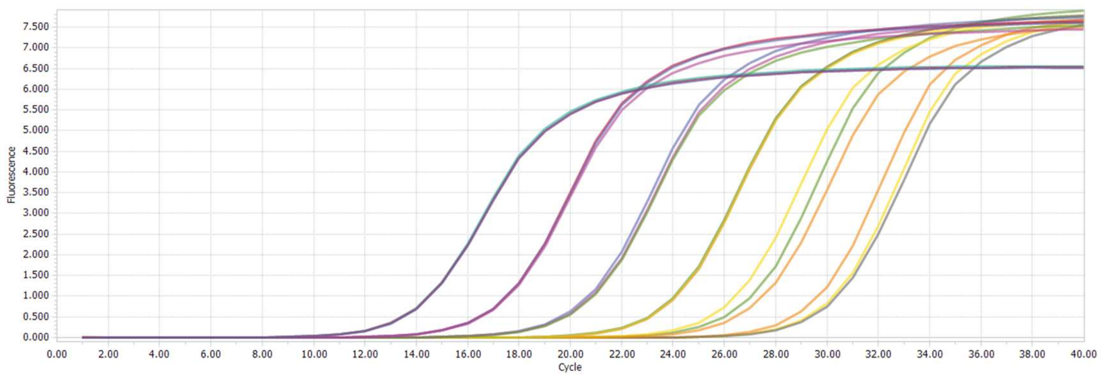

SOCS1

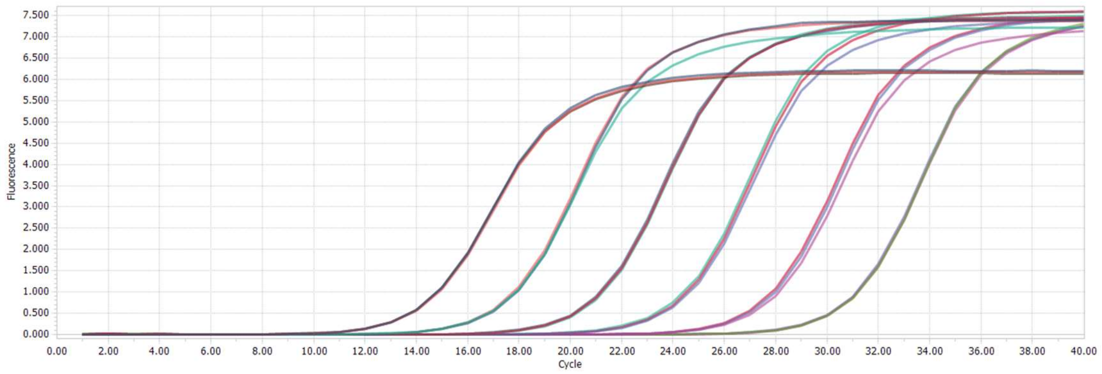
Fluorescence
Cycle
7.500
7.000
6.500
6.000
5.500
5.000
4.500
4.000
3.500
3.000
2.500
2.000
1.500
1.000
0.500
0.000
0.00
2.00
4.00
6.00
8.00
10.00
12.00
14.00
16.00
18.00
20.00
22.00
24.00
26.00
28.00
30.00
32.00
34.00
36.00
38.00
40.00

STAT6
