# Supplementary material for: Corilagin Counteracts IL-13Rα1 Signaling Pathway in Macrophages to Mitigate Schistosome Egg-Induced Hepatic Fibrosis
Source: Front Cell Infect Microbiol. 2017 Oct 18;7:443. doi: 10.3389/fcimb.2017.00443 (PMC5651236; doi:10.3389/fcimb.2017.00443)
Supplement: Supplementary file 2 [file DataSheet2.DOCX]

**Chemicals and reagents**

Corilagin (purity>99%) for the cell-based assays was purchased from National Institutes for Food and Drug Control (Beijing, China), catalogue number 111623. Corilagin for the animal assays was purchased from Chengdu PureChem-Standard Co., Ltd. (Chengdu, China), catalogue number 23094-69-1. Praziquantel was provided by the Schistosomiasis Control Institute of Hubei Province (Wuhan, China). Pentobarbital sodium was purchased from Sigma-Aldrich (Shanghai, China), catalogue number 11715. RPMI Medium 1640 basic was purchased from Gibco (Grand Island, NY, USA), catalogue number 11875-093. Foetal bovine serum (FBS) was purchased from Gibco (Grand Island, NY, USA), catalogue number 10082139. Recombinant interleukin 13 (IL-13) was purchased from Peprotech (Rocky Hill, NJ, USA), catalogue number 400-16. Cell Counting Kit-8 (CCK-8) was purchased from Dojindo 112 Laboratories (Japan), catalogue number CK04. Rabbit anti-mouse PPARγ was obtained from Proteintech Company (Wuhan, China), catalogue number 16643-1-AP. Rabbit anti-mouse KLF4 was obtained from Santa Cruz Biotechnology Company (Dallas, TX, US), catalogue number sc-20691. Rabbit anti-mouse SOCS1 was obtained from Abclonal Technology (Woburn, MA, US), catalogue number A7754. Rabbit anti-mouse p-STAT6 was obtained from Cell Signalling Technology (CST, Boston, MA, USA), catalogue number ab54461. Rabbit anti-mouse CD206 antibody was purchased from Proteintech Group, INC (Chicago, USA), catalogue number 18704-1-AP. Rabbit anti-mouse GAPDH was obtained from Wuhan Boster Biotechnology Co., Ltd (Wuhan, China), catalogue number BM1985. Biotin-conjugated goat anti rabbit IgG and streptavidin-horseradish peroxidase (HRP) conjugate were obtained from Wuhan Boster Biotechnology Co., Ltd (Wuhan, China), catalogue number BA1054. RNAiso Plus, PrimeScript™ RT reagent Kit was purchased from TaKaRa (Dalian, China), catalogue number 3733. SYBR Premix Ex Taq kit were purchased from TaKaRa (Dalian, China), catalogue number RR420A. The mouse TGF-β ELISA kits were purchased from Elabscience (Wuhan, China), catalogue number E-EL-M0051. The IL-13Rα1 and negative-control lentiviral vectors were constructed by GeneChem Co, Ltd (Shanghai, China). The IL-13Rα1 siRNA was constructed by Rebobio Company (Guangzhou, China). Lipofectamine™ 2000 was purchased from Invitrogen Company, catalogue number 11668019. *Oncomelania hupensis* was purchased from the Nanjing Center for Disease Control and Prevention (Nanjing, China).
